# Protein and microRNA biomarkers from lavage, urine, and serum in military personnel evaluated for dyspnea

**DOI:** 10.1186/1755-8794-7-58

**Published:** 2014-10-05

**Authors:** Joseph N Brown, Heather M Brewer, Carrie D Nicora, Karl K Weitz, Michael J Morris, Andrew J Skabelund, Joshua N Adkins, Richard D Smith, Ji-Hoon Cho, Richard Gelinas

**Affiliations:** 1 Biological Sciences Division, Pacific Northwest National Laboratories, Richland, WA 99354, USA; 2 San Antonio Military Medical Center, Fort Sam Houston, San Antonio, TX 78234, USA; 3 Institute for Systems Biology, Seattle, WA 98109, USA

**Keywords:** Dyspnea, Biomarkers, Lavage fluid, Serum, Urine, Proteomics, MicroRNAs

## Abstract

**Background:**

We have identified candidate protein and microRNA (miRNA) biomarkers for dyspnea by studying serum, lavage fluid, and urine from military personnel who reported serious respiratory symptoms after they were deployed to Iraq or Afghanistan.

**Methods:**

Forty-seven soldiers with the complaint of dyspnea who enrolled in the STudy of **A**ctive Duty Military Personnel for Environmental Dust Exposure (STAMPEDE) underwent comprehensive pulmonary evaluations at the San Antonio Military Medical Center. The evaluation included fiber-optic bronchoscopy with bronchoalveolar lavage. The clinical findings from the STAMPEDE subjects pointed to seven general underlying diagnoses or findings including airway hyperreactivity, asthma, low diffusivity of carbon monoxide, and abnormal cell counts. The largest category was undiagnosed. As an exploratory study, not a classification study, we profiled proteins or miRNAs in lavage fluid, serum, or urine in this group to look for any underlying molecular patterns that might lead to biomarkers. Proteins in lavage fluid and urine were identified by accurate mass tag (database-driven) proteomics methods while miRNAs were profiled by a hybridization assay applied to serum, urine, and lavage fluid.

**Results:**

Over seventy differentially expressed proteins were reliably identified both from lavage and from urine in forty-eight dyspnea subjects compared to fifteen controls with no known lung disorder. Six of these proteins were detected both in urine and lavage. One group of subjects was distinguished from controls by expressing a characteristic group of proteins. A related group of dyspnea subjects expressed a unique group of miRNAs that included one miRNA that was differentially overexpressed in all three fluids studied. The levels of several miRNAs also showed modest but direct associations with several standard clinical measures of lung health such as forced vital capacity or gas exchange efficiency.

**Conclusions:**

Candidate proteins and miRNAs associated with the general diagnosis of dyspnea have been identified in subjects with differing medical diagnoses. Since these markers can be measured in readily obtained clinical samples, further studies are possible that test the value of these findings in more formal classification or case–control studies in much larger cohorts of subjects with specific lung diseases such as asthma, emphysema, or some other well-defined lung disease.

## Background

Over 2.5 million military personnel have served in Southwest Asia since 2002 to the present, as part of Operation Iraqi Freedom, Operation Enduring Freedom, or more recently Operation New Dawn. The majority of these people were regularly exposed to geologic dust or other airborne particulate matter or toxic substances. After deployment overseas, some military personnel have reported new respiratory symptoms generally described as dyspnea that required further medical evaluation. The ‘STudy of Active Duty Military Personnel for Environmental Deployment Exposure’ (STAMPEDE) was sanctioned by the U.S. Department of Defense explicitly to evaluate military personnel who have recurrent or persistent dyspnea after deployment [1]. Our goal was to work with the STAMPEDE project to discover molecular signatures or objective biomarkers of lung disease in these individuals.

Ours was an exploratory project, since no underlying disease had been diagnosed in the STAMPEDE subjects. Biomarkers have been reported for dyspnea, but only when it is secondary to heart failure, myocardial infarction, or pulmonary infarction [2] all of which had been excluded from the STAMPEDE subjects. Even though biomarkers have been reported for different forms of asthma [3-5] and specific patterns of miRNAs and proteins have been reported for chronic obstructive pulmonary disease [6,7] or idiopathic pulmonary fibrosis [8] an assay based only on these markers might miss other lung diseases. We also decided to test urine since it has been studied as a source for protein markers of kidney diseases [9,10] or prostate cancer [11,12]. Urine miRNAs have been reported as biomarkers of polycystic kidney disease [13]. Since the rationale for STAMPEDE was to establish a diagnosis, the novelty of our approach was to profile both proteins and miRNAs in samples of BAL, urine, and serum from a group of soldiers whose dyspnea was worsening, but where heart disease was not suspected.

By comparing subjects who self-reported with dyspnea to control individuals, we established protein profiles from BAL fluid and urine. We also profiled miRNAs in samples of BAL, urine, and serum. Both protein and miRNA profiles from bronchoalveolar lavage BAL readily classified most subjects with dyspnea from controls. We also identified subsets of STAMPEDE subjects with groupings of differentially expressed proteins or miRNAs, which would be consistent with a shared underlying pathology and a common diagnosis related to asthma or possibly an obstructive lung disease. Some differentially expressed miRNAs directly correlated with abnormal measures of lung function, as recorded on their medical charts. We also found that some differentially expressed protein or miRNAs in subjects with dyspnea that were detected in BAL were also detected in urine or serum or in all three fluids.

The molecular profiles we established from the BAL samples from the fifteen control individuals begin to define the state of the normal lung. These protein and miRNA biomarkers may be valuable as a general reference for a normal, healthy lung since the healthy lung profiles represent lung ‘wellness’ at least for these individuals. Biomarkers for healthy lungs is of broader interest in the context of personalized medicine and also for judging progressive changes in the status of the lung after surgery, recovery from a disease, or treatment with a drug. This was an exploratory study. The ultimate goal, after testing the candidate markers presented here in a larger, rigorous case–control classification study in patients with a well-defined type of lung disease will be to define valid markers that help with lung disease diagnosis, disease progression, or response to drug therapy.

## Methods

### Ethics statement

The project was conducted in compliance with U.S. 32 CFR 219 (“Common Rule”) as administered within the United States Department of Defense (DoD). The Common Rule is derived from the basic ethical principles espoused in the Belmont Report (http://ohsr.od.nih.gov/guidelines/belmont.html). This project utilized de-identified BAL fluid, urine, and serum samples acquired by military researchers at San Antonio Military Medical Center. Written informed consent was obtained in advance from all study participants. The use of these samples was approved at San Antonio Military Medical Center under IRB protocol 363715. Participation in this research by the Institute for Systems Biology under protocol 2012.0007 which was also approved by the Western Institutional Review Board (Registration #00000533). The United States Army Medical Research and Materiel Command Human Research Protection Office concurred with the determination of the Western Institutional Review Board.

### Study participants

Soldiers with post-deployment respiratory symptoms were referred to the STAMPEDE protocol at San Antonio Military Medical Center from 2011 to 2013 [1]. Study subjects had returned from deployment to Southwest Asia within the past six months and complained of new onset respiratory symptoms, primarily dyspnea. All participants underwent a standard evaluation with full pulmonary function studies, high resolution chest CT scans, methacholine challenge testing and other testing such as laryngoscopy or cardiopulmonary exercise testing as clinically appropriate. The standard evaluation included flexible bronchoscopy from which BAL fluid was collected from the right middle lobe of the lung with 180 cc normal saline instilled (60 cc × 3). The BAL fluid was used to obtain cell counts, flow cytometry and cytokine levels and a portion was sent to the Institute for Systems Biology (ISB) for miRNA profiling and to Pacific Northwest National Labs (PNNL) for proteomics profiling. The morning urine was collected in a sterile specimen collection container on the day of the bronchoscopy. Samples were then placed on ice prior to centrifugation at 1500 RCF for 10 minutes at 4°C. After centrifugation, the sample was transferred to 3 cryovials and then stored at −80°C before analysis. Two Greiner Bio-One Vacuette red top with clot activator (RN 456089) collection tubes are used to collect blood on the same day as the bronchoscopy procedure. Once collected, the tubes were inverted 5 to 6 times to mix the clot activator and blood before a 30–45 minute incubation period at room temperature with the tubes in an upright positon. After clotting, the tubes were centrifuged for 10 minutes at 1200 RCF and 4°C. After centrifugation the supernatant serum was transferred into 2 cryovials and then stored at −80°C before analysis.

BAL and urine proteins were analyzed at PNNL. Serum proteins were not profiled because the added cost and complexity was beyond the scope of our effort. MiRNAs from BAL, urine, and serum were profiled at ISB. Basic demographic information about the STAMPEDE subjects was summarized [1] while information for the control subjects is summarized in Additional file 1 and Table 1.

**Table 1:** Subject numbers and clinical diagnoses

**Subject number**	**Clinical finding**	**BAL miRNA group**
1	Undiagnosed	
2	Undiagnosed	
3	Undiagnosed	
4	Undiagnosed	
5	Airway Hyperreactivity	
6	Asthma	
7	Undiagnosed	
8	Asthma	
9	Undiagnosed	
10	Airway Hyperreactivity	
11	Undiagnosed	**1**
12	Obstruction	**1**
13	Low DLCO	**1**
14	Air Trapping	**1**
15	constrictive bronchiolitis	**1**
16	Low DLCO	**1**
17	Asthma	**1**
18	Undiagnosed	**1**
19	Airway Hyperreactivity	
20	Abnormal Cell Count	
21	Airway Hyperreactivity	**1**
22	Asthma	
23	COPD	**1**
24	Abnormal Cell Count	
25	Abnormal Cell Count	
26	Airway Hyperreactivity	
27	Airway Hyperreactivity	
28	Asthma	**2**
29	Lung Nodule	
30	Abnormal Cell Count	
31	Undiagnosed	
32	Isolated low DLCO	
33	Inhalation Injury	
34	Asthma	**2**
35	Negative	**2**
36	Undiagnosed	**2**
37	Abnormal Cell Count	
38	Asthma	
39	asthma	
40	Asthma/VCD	
41	Undiagnosed	
42	Excessive dynamic airway collapse	
43	GERD/AHR	
44	Abnormal Cell Count	**2**
45	Low DLCO	**2**
46	Undiagnosed	
47	Airway Hyperreactivity	
48	Undiagnosed	

Legend: DLCO – diffusing capacity for carbon monoxide; GERD/AHR – gastroesophageal reflux with secondary airway hyperreactivity; VCD – vocal cord dysfunction.

### BAL sample preparation for proteome analysis

Samples (ranging from 0.46 to 1.90 mL) were thawed, desalted, and concentrated with Amicon 3 K MWCO spin filters (EMD Millipore, Billerica, MA). First, the filters were washed with 4 mL of 100 mM NH4HCO3 (buffer) and centrifuged at 4,000 × g for 40 minutes at 4°C. Next, the samples were applied to the filters and buffer was added to adjust the volume to 4 mL, then centrifuged at 4000 × g, 4°C for 45 minutes. Samples were washed by filling the filter portion with 4 mL of buffer (ensuring resuspension of the sample from the bottom of the filter) and then centrifuged again for 75 minutes to ensure the dead volume was reached. The samples were transferred from the filter portion of each concentrator to a 2.0 mL microcentrifuge tube. The filters were rinsed by adding 100 μL of buffer, vortexing briefly and then using a pipet tip to “wash” the two membranes 3× each. Then the wash sample was combined with the main sample. Next, the volume of each sample was measured and normalized (adjusted) to match the largest volume of the set of samples being processed together. The protein concentration of each sample was calculated by a Bicinchoninic (BCA) assay. Urea and dithiothreitol (DTT) were added from stock solutions to final concentrations of ~8 M and 5 mM, respectively and the samples were reduced and denatured at 60°C for 30 minutes. Iodoacetamide was added to 40 mM and samples were incubated at 37°C for 1 hour to alkylate. The samples were diluted eight-fold with buffer, and CaCl_2_ was added to 1 mM. Samples were digested with trypsin (Affymetrix/USB; Santa Clara, CA) in a 1:50 (w:w) ratio of trypsin to protein at 37°C for 3 hours. Samples were purified on C18 solid phase extraction ‘Discovery’ columns (Supelco-Sigma-Aldrich, Bellefonte, PA) followed by concentration, assaying peptide concentration using the BCA protein assay (Thermo Scientific Pierce; Rockford, IL), and diluted to 0.5 μg/μL for mass spectrometry (MS) analysis.

### Urine sample preparation for proteome analysis

The urine samples were processed using an epMotion device (Eppendorf, Hauppauge, NY) which was used to automatically pipette and process samples: 500 μl of each urine sample was loaded into a 1.0 mL 96-well plate and concentrated to dryness. Next, 107 μL of liquid 8 M urea was added to the dried urine, vortexed, and then briefly centrifuged. After a 50-fold dilution the protein concentration was assayed by the BCA procedure, followed by addition of DTT to 8.3 mM. The plate was vortexed, centrifuged briefly, and incubated for 1 hour with shaking. Iodoacetamide was added to 36 mM and the plate was incubated at 37°C for 1 hour in the dark with shaking. The samples were then diluted 8-fold with 100 mM NH_4_HCO_3_ (buffer), CaCl_2_ was added to 1 mM, and trypsin was added in a 1:50 trypsin:protein (w:w) ratio. The plate was incubated at 37°C for 3 hours with shaking. Samples were purified as described above on C18 columns (Agilent, Santa Clara, CA), concentrated, re-assayed for protein concentration, and diluted to 0.3 ug/uL for MS analysis.

### Reversed phase liquid chromatography (RPLC) separation and MS(/MS) acquisition

The liquid chromatography (LC) system was custom built at PNNL using two Agilent 1200 nanoflow pumps and one Isco constant pressure capillary pump (Teledyne-Isco, Lincoln, NE), various Valco valves (Valco Instruments Co., Houston, TX), and a PAL autosampler (Leap Technologies, Carrboro, NC). Reversed-phase columns were prepared in-house by slurry packing 3-μm Jupiter C18 (Phenomenex, Torrence, CA) into 35-cm × 360 μm o.d. × 75 μm i.d fused silica (Polymicro Technologies Inc., Phoenix, AZ) using a 1-cm sol–gel frit for media retention (unpublished PNNL variation of the method of Maiolica et al. [14]. Trapping columns were similar but used a 4 cm length of 100 μm i.d. fused silica that was fritted on both ends. Mobile phases consisted of 0.1% formic acid in water (A) and 0.1% formic acid acetonitrile (B) operated at 300 nL/min with a gradient profile as follows (min: %B); 0:5, 2:8, 20:12, 70:35, 97:60, 100: 95. Sample injection occurred 40 min prior to beginning the gradient while data acquisition lagged the gradient start and end times by 10 min to account for column dead volume that allowed for the tightest overlap possible in two-column operation. Two-column operation also allowed for columns to be ‘washed’ (shortened gradients) and re-generated off-line.

MS analysis was performed using a Velos Orbitrap mass spectrometer (Thermo Scientific, San Jose, CA) outfitted with a custom electrospray ionization interface. Electrospray emitters were custom made by chemically etching 150 um o.d. × 20 um i.d. fused silica [15]. The heated capillary temperature and spray voltage were 350°C and 2.2 kV, respectively. Data was acquired for 100 min after a 10 min delay from when the gradient started. Orbitrap spectra (AGC 1 × 106) were collected from 400–2000 m/z at a resolution of 60 k followed by data-dependent HCD MS/MS (collision energy 32%, AGC 5 × 10^4^) of the ten most abundant ions, excluding single charge states. A dynamic exclusion time of 60 sec was used to discriminate against previously analyzed ions using a 0.55 to 1.55 Da mass window.

### Mass spectrometry data analysis

Accurate mass and time (AMT) tag [16-19] results were filtered for a mass error less than 3 ppm and by statistical tools for AMT tag confidence [20] for a uniqueness probability score greater than 0.5 and a false discovery rate (FDR) threshold < 10%. The Accurate Mass and Time (AMT) tag approach to proteomics we used is a peak area-based form of quantification for high-throughput proteomics. It has some advantages over labeling techniques for large samples sizes. But, compared to labeling techniques it can have a higher *peptide* false discovery rate. As such, the <10% peptide false discovery rate refers only to the complexity of this specific AMT Tag database which is simply based on mass and elution times. But since we used multiple unique peptides for protein identifications, the overall *protein* false discovery rate is much lower than the peptide false discovery rate [17,21]. The resulting datasets were log2 transformed. Potential outlier datasets were identified using robust Mahalanobis distance squared values associated with the peptide abundance vector (rMd-PAV) and a p-value threshold less than 0.001 as recommended by the developers of the algorithm [22]. The optimal normalization algorithm was determined by ‘Statistical procedure for the analysis of peptide abundance normalization strategies’ (SPANS: [20]) to be a mean center with the rank invariant peptide (RIP) selection having a *p*-value threshold of 0.1 for the BAL and a mean center with the top *L* Order Statistics (LOS) peptide selection having a *p*-value threshold of 0.05 for the urine datasets. The correlation scores were summarized between datasets derived from different individuals as shown (Additional file 2). A probabilistic principal component analysis, within the pcaMethods package version 1.50.0(4) in R version (3.0.0; 64-bit), was performed on the datasets containing missing values (Additional file 3). The enrichment analysis of GO biological processes terms for key groups of proteins was performed using the Database for Annotation, Visualization and Integrated Discovery (DAVID, ver 6.7) software [23].

### Statistical analysis

Hypothesis tests were performed with MSstats version 1.0.0 [24], with missing-action set to remove, to determine statistical differences in protein abundance between control and disease samples. *P*-values were corrected for multiple comparisons using the Benjamini-Hochberg *p*-value adjustment [25]. For heatmaps, protein abundance vectors were arranged by ascending fold-difference. All statistical tests were performed with R [26]. The net result of the removal of outliers and the application of the filtering steps described above was to identify 79 proteins derived from BAL and 74 proteins from urine that were differentially expressed in the dyspnea subjects and controls as shown in the analysis figures, below. The overlap rate for proteins between different subjects is given on the heatmaps, below or in histograms (data not shown). The BAL and urine protein abundances followed approximately normal distributions as shown by quantile plots (Additional file 4).

### MicroRNA analysis

RNA enriched for miRNA was isolated from 250 μL aliquots of bronchial alveolar lavage fluid, 150 μL aliquots of serum, and 250 μL aliquots of urine from STAMPEDE subjects (n = 47) or control individuals (n = 15) by using the miRNeasy mini kit (Qiagen, cat. 217 004). The yields of total cell RNA from these samples were typically 30 nanograms or lower. The concentrations of about 800 human miRNAs were determined by using the NanoString nCounter human miRNA expression assay kit version 2.1 following the manufacturer’s instructions (NanoString Technologies, Seattle WA). This quantitation method is based on hybridization of a miRNA to complementary oligonucleotides that also carry functional groups for purification and fluorescent tags for detection [27]. Several samples from urine, serum, or lavage were analyzed more than once which let us assess the reproducibility of the RNA isolation and the NanoString profiles. After normalization against a global mean and correction for background (greater than 50 counts), typical mean miRNA levels from two replicate experiments (serum, BAL) or three replicates (urine) were plotted (Additional file 5). Standard errors of the mean were typically less than 10% of the mean. Selected miRNA levels measured by NanoString profiling were verified in a subset of STAMPEDE subjects and controls by RT-PCR studies. RNA was isolated from lavage as described above, and reverse transcribed (Qiagen miScript II RT kit, cat. 218161). Primers specific for miRNAs 187-3p, 371a-5p, 212-3p, 1915-3p, 4516, 320e, and 630 were purchased from Qiagen and quantitative RT-PCR was completed as described [28]. The RT-PCR results substantially confirmed the NanoString levels for several differentially expressed miRNAs that helped classify one sub-set of dyspnea subjects (Additional file 6).

For identification of sub-groups by cluster analysis or correlation analysis, the NanoString data for replicate measurements (about 1/3 of all samples) were averaged. The data was then normalized by a factor derived from the geometric mean of the positive controls. Next, a background correction was applied, which was based on the mean plus two standard deviations of the negative controls. Finally, the data was again normalized using a factor calculated from the geometric mean of the highly abundant probes. These steps were performed using the R package *NanoStringNorm*[29]. Next, the normalized, background corrected data was log2-transformed. Then, using the Bioconductor package ‘LIMMA’ [30], differentially expressed miRNAs between the STAMPEDE and control groups we identified. P-values from the moderated t-test and fold-changes between groups were obtained and differentially expressed miRNAs were identified by the criteria of p-values less than 0.01 and fold-changes greater than ±2-fold. Pearson correlation coefficients were calculated between several numerically continuous medical chart parameters and selected miRNA values.

## Results

### Proteomics analysis of lung fluid

**Figure 1:**
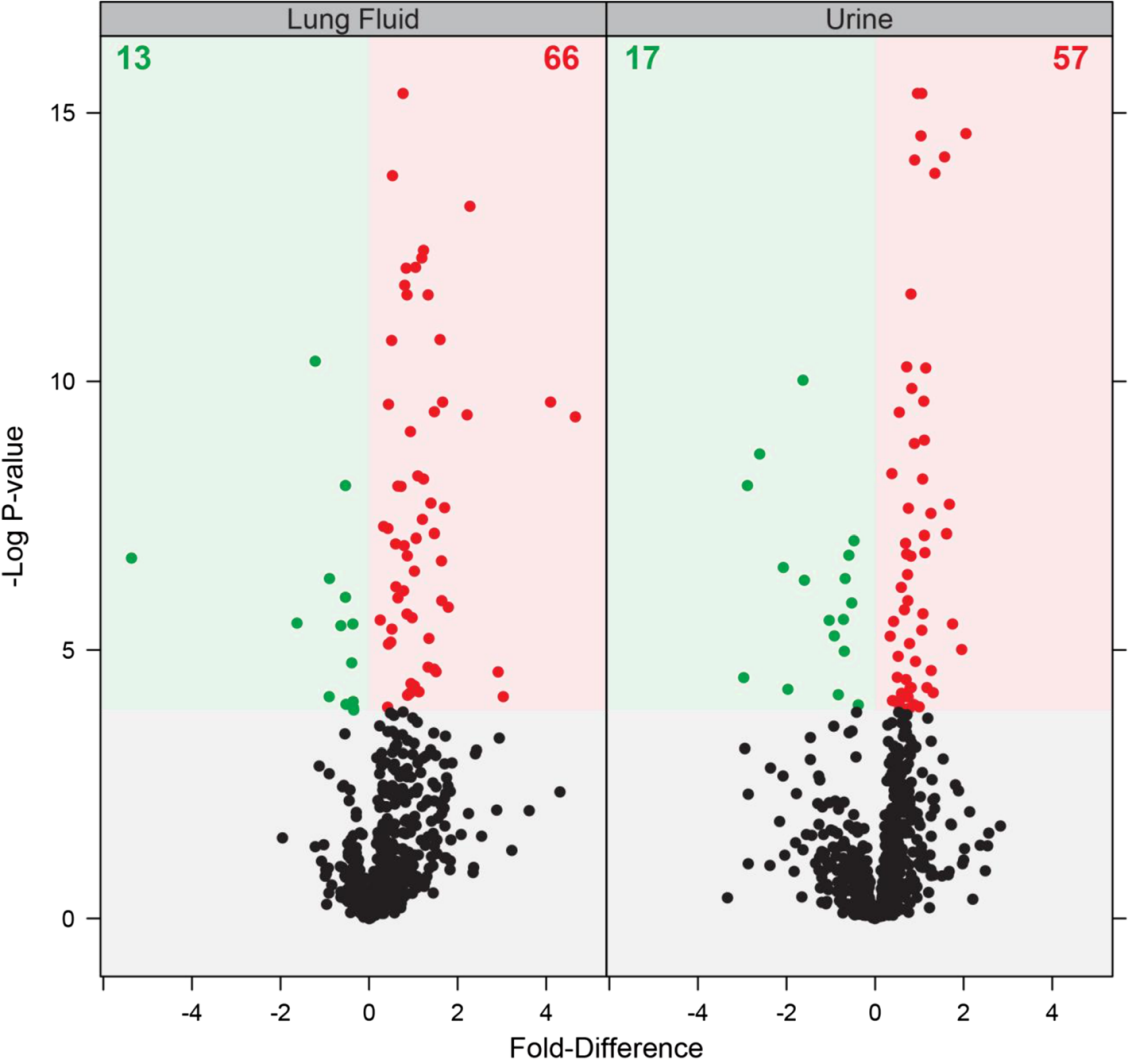
**Scatterplot of lung fluid (left) and urine (right) proteins.** Significant proteins have an adjusted p-value less than 0.05. Proteins with significantly greater and lower abundance in disease are shown in red and green, respectively, with the actual number displayed at top of plot.

Lung fluid was obtained by performing bronchoalveolar lavage on 15 control and 47 STAMPEDE subjects with dyspnea. Proteins were extracted and prepared for analysis by liquid chromatography coupled with online high resolution mass spectrometry and low resolution tandem mass spectrometry (LC-MS(/MS)). An AMT tag approach was used to analyze the datasets produced by the mass spectrometer. An rMd-PAVS analysis [22] identified outliers with a p-value < 0.001, 2 controls and 7 disease samples, which were removed from the analysis. Subsequent comparisons were between 40 dyspnea and 13 control profiles. The analysis identified 12,340 unique peptides corresponding to 987 proteins. A SPANS analysis [20] based on an in-house developed R application [26] was used to determine the optimal normalization for the peptide abundance values. The optimal normalization was mean-centered, using rank invariant selected peptides with a p-value > 0.10. An MSstats analysis [22] was performed removing proteins with insufficient observations to perform the hypothesis test. The removal of proteins with insufficient observations resulted in 652 proteins, of which 79 (~12%) showed a significant difference in abundance (*p*-value < 0.05) between control and disease (66 proteins significantly greater and 13 significantly lower in disease compared to control). The effects of these filters on the data are illustrated by the scatter plot presented in Figure 1. The 79 proteins were further analyzed by one-dimensional cluster analysis across all informative study subjects which were readily distinguished from controls as shown in Additional file 7. The 79 BAL proteins are listed by their UniProt identifiers in Additional file 8. All were used to search for GO terms. A sub-set of these proteins returned gene ontogeny (GO) terms (p values <0.01) for digestion (ACE, TRY6, TFF3, AMY2B), sterol or cholesterol or lipid homeostasis (APOA1, APOA2, FABP4), and the proteins APOA1 and APOA2 also returned terms for negative regulation of very-low-density lipoprotein particle remodeling; regulation of cytokine secretion during immune response; and negative regulation of cytokine secretion during immune response as summarized in Additional file 9. These GO terms are generally consistent with tissue repair or remodeling or leakage of the blood into the lung.

### Proteomic analysis of urine samples

Proteins were extracted from urine obtained from 15 controls and 48 dyspnea individuals and analyzed by LC-MS(/MS) as described above. An rMd-PAVS analysis [22] identified outliers, 3 samples from the disease group, which were removed from the analysis, leaving a total of 45 samples in the dyspnea group. These dyspnea profiles were compared to the 15 control urine profiles. The analysis identified 9,330 unique peptides corresponding to 846 proteins. Peptide abundances were normalized and proteins with insufficient observations were removed as described above. The optimal normalization was median-centered, using the top *L* Order Statistics peptide selection, with *L* being 614. The effects of these filters on the urine data are illustrated by the volcano plot presented in Figure 1. The removal of proteins with insufficient observations resulted in 695 proteins, which 74 (~11%) showed a significant difference in abundance (*p*-value < 0.05) between control and disease (57 proteins significantly greater and 17 significantly lower in the dyspnea group compared to control). Differentially expressed proteins derived from urine readily distinguished dyspnea subjects from controls (Additional file 10). The 74 proteins differentially expressed in urine are listed by their UniProt identifiers in Additional file 8. A sub-set of these proteins returned GO terms (p-values <0.06) for cell adhesion (AMBP, PVR, WISP2, CADM4, CD44, CD99); regulation of growth (WISP2, CD44, CSF1, VGF); regulation of cell migration, locomotion, or cell motion (CSF1, ROBO4, THY1); wound healing (HMCN1, CD44, CD59) all of which are again generally consistent with tissue remodeling (Additional file 9).

### Protein found in BAL and urine samples

**Figure 2:**
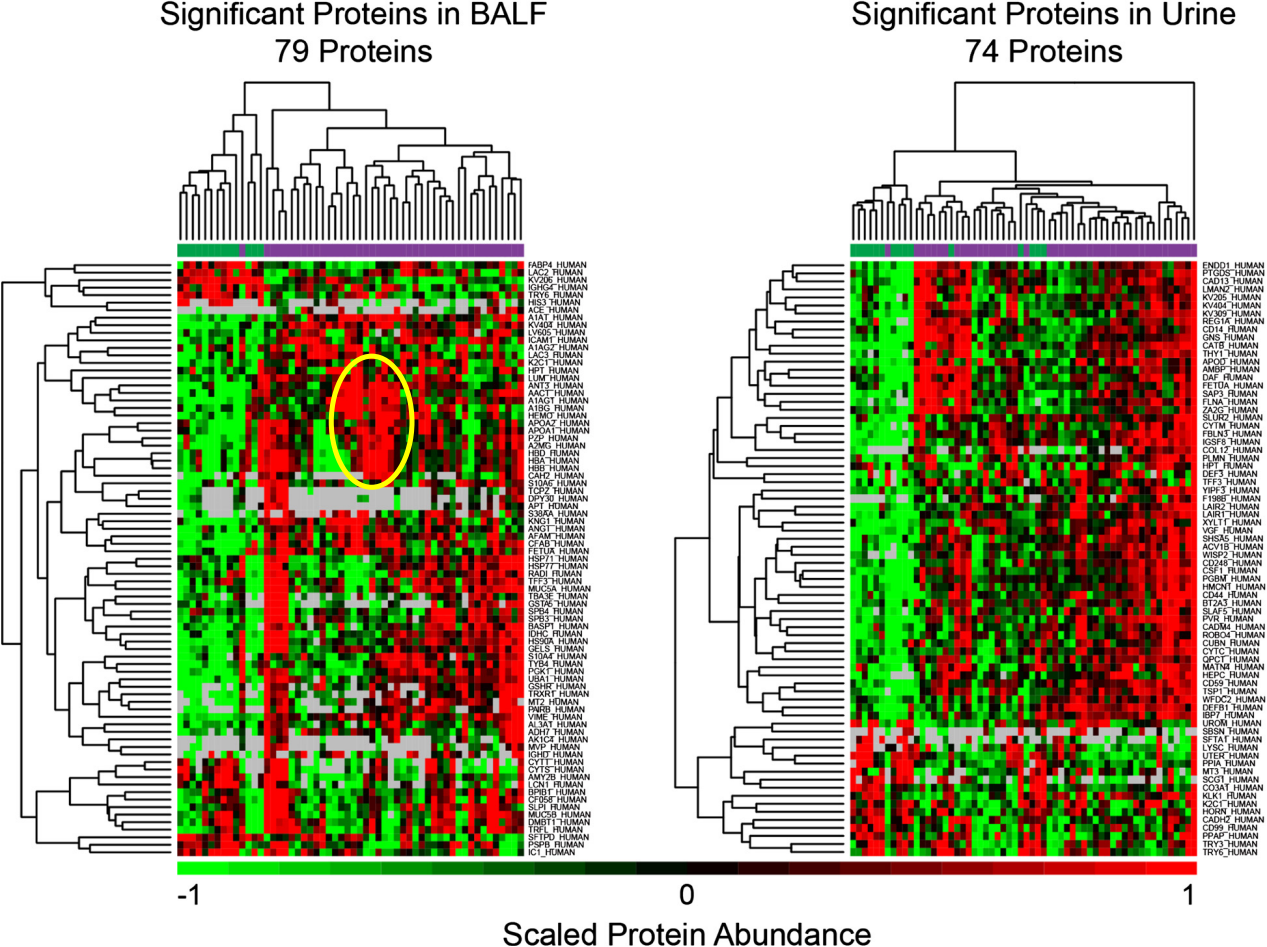
**Unsupervised cluster analysis of BAL and urine proteins.** BAL proteins from Additional file 7 and urine proteins from Additional file 10 were re-clustered for control and STAMPEDE subjects. Protein abundances were scaled by z-score, with green and red representing 1 standard deviation below and above the mean, respectively. The bar above the heatmap indicates controls (green) and disease (purple) subjects. The oval identified a subset of subjects who expressed a group of proteins in common, as discussed in the text. Gray indicates that a protein was not detected for that individual. Some individuals have multiple columns (e.g. D14 in 3B) which resulted from technical replicate analyses. These technical replicates clustered together.

Six proteins TRY6, KV404, K2C1, HPT, FETUA, and TFF3 were differentially expressed in STAMPEDE subjects relative to controls and appeared in both in BAL and urine. All six of these were generally higher in BAL or urine from dyspnea subjects, except for K2C1 and TRY6 which were higher in control urine rather than urine from dyspnea subjects. That these proteins were detected in both fluids, at least raises the possibility that they could have originated in the lung, passed into the blood stream, and were excreted in the urine (Additional file 8). After unsupervised cluster analysis of the BAL proteins shown in Figure 2, a sub-set of dyspnea subjects that co-expressed a group of proteins was recognized. This group, designated with the yellow oval in Figure 2 includes subjects 21, 13, 46, 15, 9, 25, 17, 19, 36, and 35. The mean expression levels of these thirteen proteins in the controls, the STAMPEDE subjects and the STAMPEDE sub-set mentioned above along with other statistical parameters and their common names are listed Tables 2 and 3. The proteins expressed in this STAMPEDE sub-group include many commonly found in blood plasma (A2MG, APOA2, APOA1) or in red blood cells (HBA and HBB) which would be consistent with bleeding or tissue remodeling (or conceivably, the bronchoscopy procedure itself). Five of these ten subjects (13, 15, 17, 19 and 21) also expressed a common set of miRNAs (see below) as might be expected if there were a common underlying pathology in the lung. Other subject groups may also be recognized, for example, by close inspection of the dendrograms that group the samples (Figure 2). The six proteins that were detected in both urine and lavage as well as the thirteen proteins detected in BAL fluid represent the best candidates for further validation studies.

**Table 2:** **Proteins upregulated in BAL from controls, STAMPEDE subjects and a subset STAMPEDE subjects (21, 13, 46, 15, 9, 25, 17, 19, 36, 35), that were identified after cluster analysis (Figure **2**)**

	**Control subjects**			**STAMPEDE subjects**			**Subset of STAMPEDE subjects**			**STAMPEDE vs CONTROLS**	**STAMPEDE subset vs controls**
**Entrez ID**	Mean	ST DEV	CV, %	Mean	ST DEV	CV, %	Mean	ST DEV	CV, %	p value	p value
**A1AG1**	18.69	0.40	2.1	19.49	0.52	2.7	19.98	0.44	2.2	0.00055429	5.95509E-07
**A1BG**	18.95	0.32	1.7	19.20	0.33	1.7	19.52	0.13	0.6	0.0001927	4.63441E-05
**AACT**	17.34	0.50	2.9	17.99	0.35	2.0	18.37	0.13	0.7	0.0010359	6.39218E-06
**ANT3**	17.62	0.39	2.2	18.15	0.35	1.9	18.50	0.17	0.9	0.00037815	3.80627E-06
**HEMO**	18.74	0.29	1.5	19.07	0.31	1.6	19.37	0.27	1.4	2.7297E-05	0.000126029
**APOA1**	17.42	1.22	7.0	18.53	0.91	4.9	19.10	0.52	2.7	9.4027E-05	0.000842961
**APOA2**	15.72	1.26	8.0	16.65	1.18	7.1	17.09	1.08	6.3	0.00057807	0.018670507
**PZP**	17.01	0.99	5.8	17.86	0.71	4.0	18.27	0.57	3.1	3.3055E-05	0.003711976
**A2MG**	16.74	0.85	5.1	17.24	0.63	3.6	17.56	0.59	3.4	1.9526E-05	0.024453179
**HBA**	16.29	1.93	11.9	17.48	1.60	9.1	18.29	1.78	9.8	1.4869E-05	0.025085649
**HBB**	15.96	1.94	12.1	17.67	1.51	8.6	18.42	1.71	9.3	7.2466E-06	0.006838885
**HBD**	16.42	2.08	12.7	17.90	1.71	9.5	18.64	2.24	12.0	5.1691E-07	0.014381261

**Table 3:** **Proteins upregulated in BAL from a subset STAMPEDE subjects (21, 13, 46, 15, 9, 25, 17, 19, 36, 35), after cluster analysis (Figure **2**)**

**BAL proteins; uniprot ID**	**Protein name**
**ANT3**	ADP/ATP translocase 3
**AACT**	Alpha-1-antichymotrypsin
**A1AG1**	Alpha-1-acid glycoprotein 1
**A1BG**	Alpha-1B-glycoprotein
**HEMO**	Heme oxygenase
**APOA2**	Apolipoprotein a-2
**APOA1**	Apolipoprotein a-1
**PZP**	Pregnancy zone protein (a protease inhibitor)
**A2MG**	Alpha-2-macroglobulin
**HBD**	Human beta defensin
**HBA**	Hemoglobin, alpha chain
**HBB**	Hemoglobin, beta chain

### MicroRNAs typically expressed in BAL fluid

MiRNAs in BAL samples were profiled from 47 STAMPEDE subjects with dyspnea and 15 control individuals with no known lung abnormalities. While the NanoString profiling system can quantitate over 800 different miRNAs, only about 50 of these were routinely detected in typical BAL samples. This is typical for some body fluids, although hundreds of miRNAs are typically found in serum and urine samples [28]. The top twenty-three miRNAs that were most frequently observed in dyspnea or control profiles are listed in Table 4, which simply presents molecular counts, after normalization. MiRNAs 1246, 1283, and 630 were found in all 62 samples (dyspnea and control) in this study, while 23 miRNAs were detected in at least 56 of 62 (90%) of the samples profiled. MiRNAs 4516, 630 and 320 were expressed in most samples at high levels, but they did not distinguish dyspnea subjects from controls as shown in Additional file 5. The levels of many miRNAs such as 4443, 143-3p, 574-5p, and 378e were unchanged between dyspnea samples and controls. These may represent miRNAs that are usually expressed in the upper airways and would be sampled by a typical bronchial lavage.

**Table 4:** Frequently expressed miRNAs in lavage fluid

**MiRNA**	**Number of positive samples (%)**	**Average counts in subjects**	**average counts in controls**	**Stdev of patient**	**Stdev of control**
**1246**	62 (100)	204.8	243.2	14.6	144.2
**1283**	62 (100)	738.0	809.8	14.1	311.3
**630**	62 (100)	2719.4	1026.8	625.6	590.4
**4516**	61 (98)	4537.7	8310.6	37.8	6731.2
**21-5p**	61 (98)	946.7	1130.3	111.7	340.2
**222-3p**	61 (98)	126.2	134.1	11.3	37.0
**25-3p**	61 (98)	223.7	202.1	13.2	92.6
**601**	61 (98)	172.6	92.6	190.3	30.8
**378e**	60 (97)	321.0	310.7	18.5	150.5
**574-5p**	60 (97)	103.2	99.4	20.9	36.5
**320e**	60 (97)	3942.6	3182.3	84.6	2620.1
**1183**	59 (95)	184.7	237.6	12.5	98.4
**598**	59 (95)	204.3	249.7	18.0	109.2
**363**	59 (95)	188.8	224.9	38.3	118.2
**143-3p**	59 (95)	184.2	179.6	11.9	75.4
**302d**	59 (95)	156.3	137.0	19.4	51.3
**4443**	59 (95)	322.9	324.4	9.5	196.4
**200a-3p**	58 (94)	134.5	157.9	16.3	57.7
**4454**	58 (94)	1130.4	882.0	26.1	651.6
**495**	57 (92)	135.0	144.4	10.2	72.6
**141-3p**	56 (90)	93.8	111.6	9.5	27.8
**761**	56 (90)	108.5	123.1	16.3	48.3
**570**	56 (90)	119.2	91.9	46.3	34.7

### Differential MiRNA expression in BAL fluid

**Figure 3:**
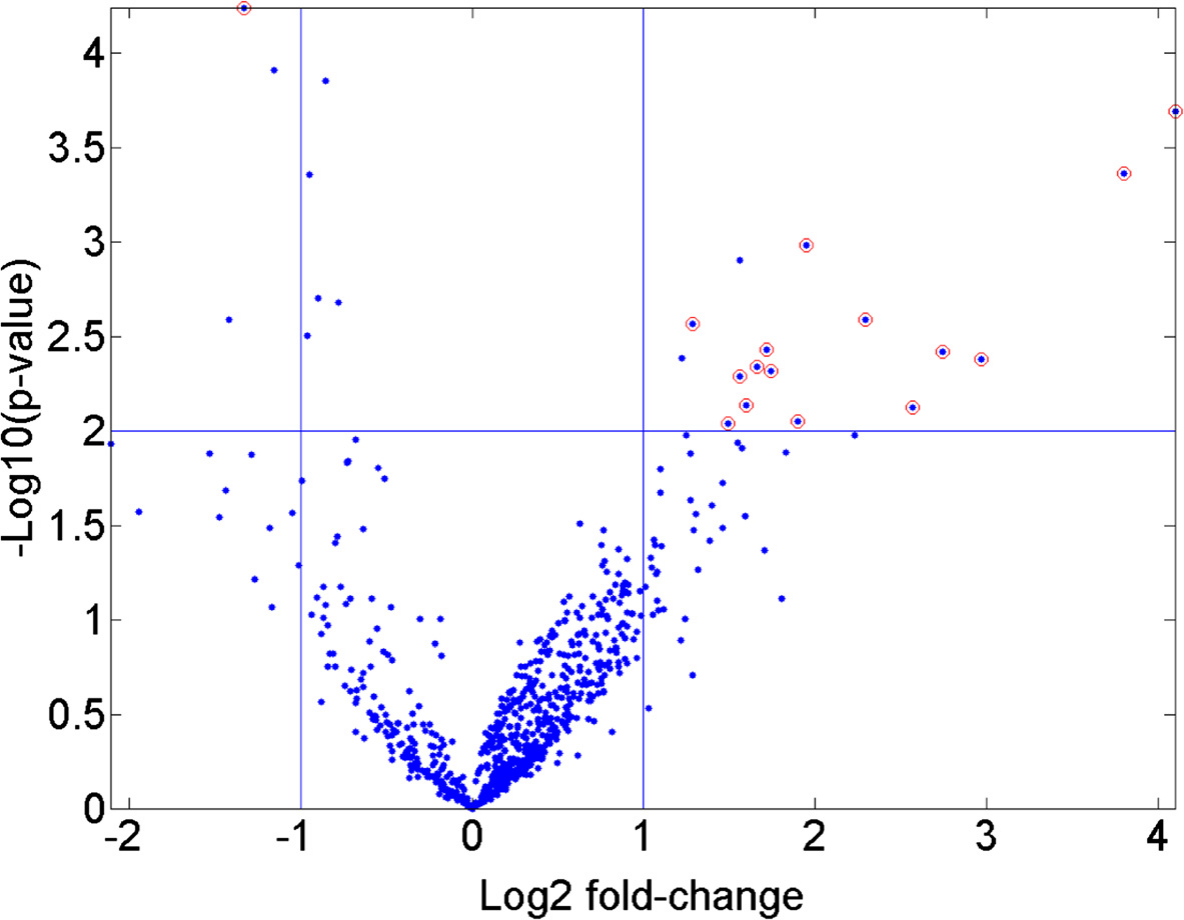
**Criteria for differentially expressed miRNAs.** The scatter plot illustrates the p-values as a function of fold-change that defined significantly differentially expressed miRNAs. Sixteen DEmiRs that met the criteria of more or less than 2-fold over- or under expressed relative to controls (log_2_ expression less than −1 or greater than +1) with p-values lower than 0.01 (−log_10_2) are shown. Similar criteria were applied for the selection of DEmiRNA from urine or serum.

**Figure 4:**
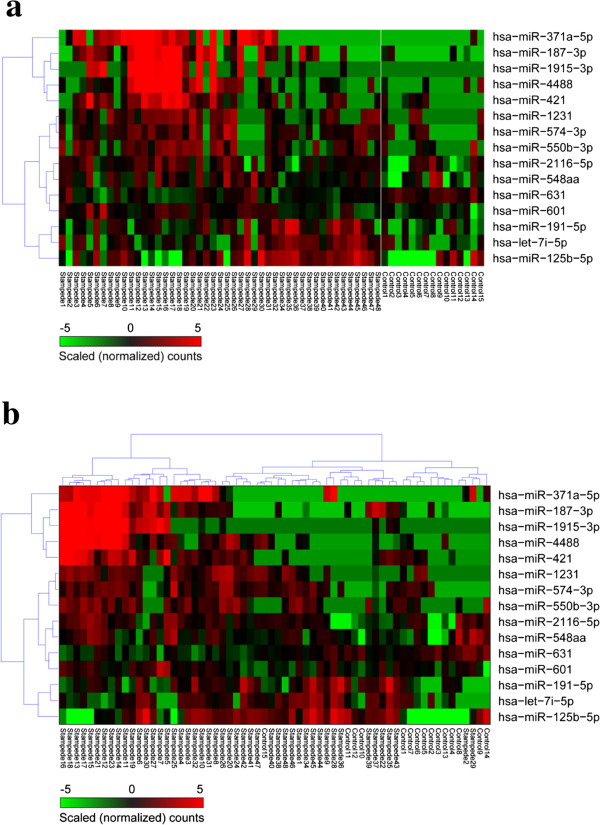
**Cluster analysis of differentially expressed miRNAs from BAL. a**. MiRNAs were clustered by their expression levels (one way clustering). **b**. Both miRNA expression and subject order were clustered to help recognize similar groupings (two-way clustering). No lavage sample was available for profiling from subject 33.

Differentially expressed miRNAs between dyspnea subjects and controls were identified in BAL, serum , and urine were defined as having a ± 2-fold expression change, a mean count greater than the global mean, and a *p*-value less than 0.01. This is represented graphically in Figure 3. Differentially expressed miRNAs selected by these criteria were displayed after one- and two-dimensional cluster analysis in Figure 4.

Two groups of dyspnea subjects could be distinguished from controls on the basis of having a shared pattern of miRNA expression. Subjects 16, 18, 13, 17, 15, 21, 12, 23, 14, & 11 defined one group and showed elevated expression of miRNAs 371a-5p, 187-3p, 1915-3p, 4488, and 421. This group is prominent in the upper left corner of Figure 4b and will be referred to hereafter as Group 1. Box plots for the difference between group 1 and the controls are given in Additional file 11. Some other subjects (19, 6, 30, 27, 7, and 5) expressed three of these five miRNAs and may be related to group 1. Likewise miRNAs 212-3p, 4532, and 489 were elevated in many group 1subjects, but these miRNAs were ultimately removed by the statistical tests applied to Figure 4. For each of these miRNAs group 1 expression is significantly higher than expression in the controls (*p* < 0.003) or in the 37 other patients in the study (*p* <0.07 or lower) as shown in Table 5.

**Table 5:** MiRNAs that define group 1 of dyspnea subjects

**MiRNA**	**Mean**	**st dev**	***p *** **vs controls**	***p *** **vs other subjects**
**187-3p**	899	567	0.0009	0.0011
**1915-3p**	442	250	0.00048	0.00055
4488*	256	113	0.00031	0.00036
371a-3p**	266	163	0.00098	0.079
**421**	110	63	0.0027	0.005
**1915-3p**	442	250	0.00048	0.00055

Legend: *p* values between the levels of the indicated miRNAs in the subjects of group 1 were calculated with the levels in the 15 controls or the subjects not in group 1 by a simple t-test. *, an outlier value from subject 16 was excluded from the calculations; **, a few other subjects expressed this miRNA at or above the mean, but did not otherwise fit the criteria for inclusion in group 1.

A second group of subjects (subjects 34, 45, 44, 28, 35, and 36) was recognized who expressed three miRNAs (191-5p, let-7i-5p, 125b) at levels higher than controls but less robustly than the miRNA expression observed in group 1. This group shows much lower expression of miRNAs 371a-5p, 187-3p, 1915-3p, 4488, 421, 663a relative to other subjects with dyspnea and the controls. These two miRNA classification patterns are distinct from one another as well as from the controls which may suggest distinct lung pathologies or clinical diagnoses.

### MiRNA expression in urine and serum

**Figure 5:**
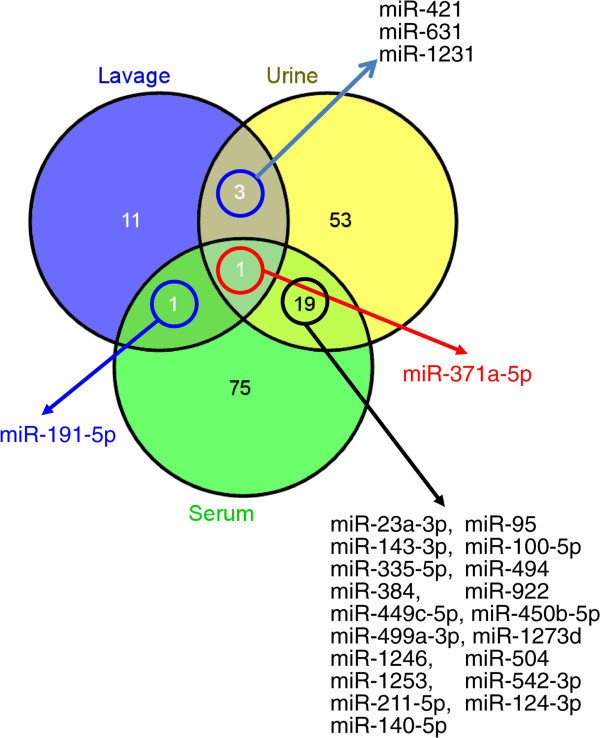
**Venn diagram that shows differentially expressed miRNAs that were shared between two or more clinical samples of BAL, serum, or urine from STAMPEDE subjects.** MiR-371a-5p and 191-5p were up-regulated in BAL and serum; in lavage and urine miR-371a-5p and miR-421, 631, and 1231 were detected; and miRNAs 371a-5p and 23a-3p, 143-3p, 335-5p, 384, 449c-5p, 499a-3p, 1246, 1253, 211-5p, 140-5p, 95, 100-5p, 494, 922, 450b-5p, 1273d, 504, 542-3p, and 124-3p were detected in both urine and serum.

**Figure 6:**
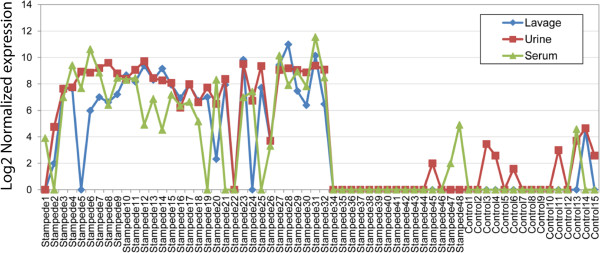
The levels of miR-371a-5p in BAL, serum, and urine in STAMPEDE subjects and controls.

MiRNAs were profiled in urine and serum from STAMPEDE subjects and controls and cluster analysis of the differentially expressed miRNAs are given in Additional files 12 and 13. Unlike the group 1 subjects that shared a profile of miRNA expression from BAL, no such pattern was obvious from the cluster analysis of urine or serum. But some miRNAs were co-expressed in two of the fluids studied and one miRNA (371a-3p) was reliably detected in all three fluids studied (Figure 5). Twenty miRNAs were reliably detected and differentially expressed in both urine and serum, while four miRNAs were expressed in both lavage and urine and two miRNAs were expressed in both lavage and serum. The elevation of mMiRNA 371a-5p expression in all three fluids was easily recognized in x-y plots of all of the expression data taken pair-wise as shown in Additional file 14, or when the normalized expression levels of this miRNA were plotted for the subjects and controls (Figure 6). MiRNAs in dyspnea subjects that are found in both urine and serum may arise in tissues or organs outside the lung as a result of the body’s response to some degree of hypoxia. A miRNA such as miR-371a-5p that is found in all three body fluids could originate in a lung tissue and pass into the blood stream (and eventually the urine) if it was derived from a wide-spread pathologic process such as tissue remodeling that included vascular leakage, and if its concentration was high enough. The miRNAs most appropriate for validation studies were those that defined groups 1 and 2 (Figure 4; Table 5).

### Specific miRNAs are associated with clinical measures of pulmonary function

**Figure 7:**
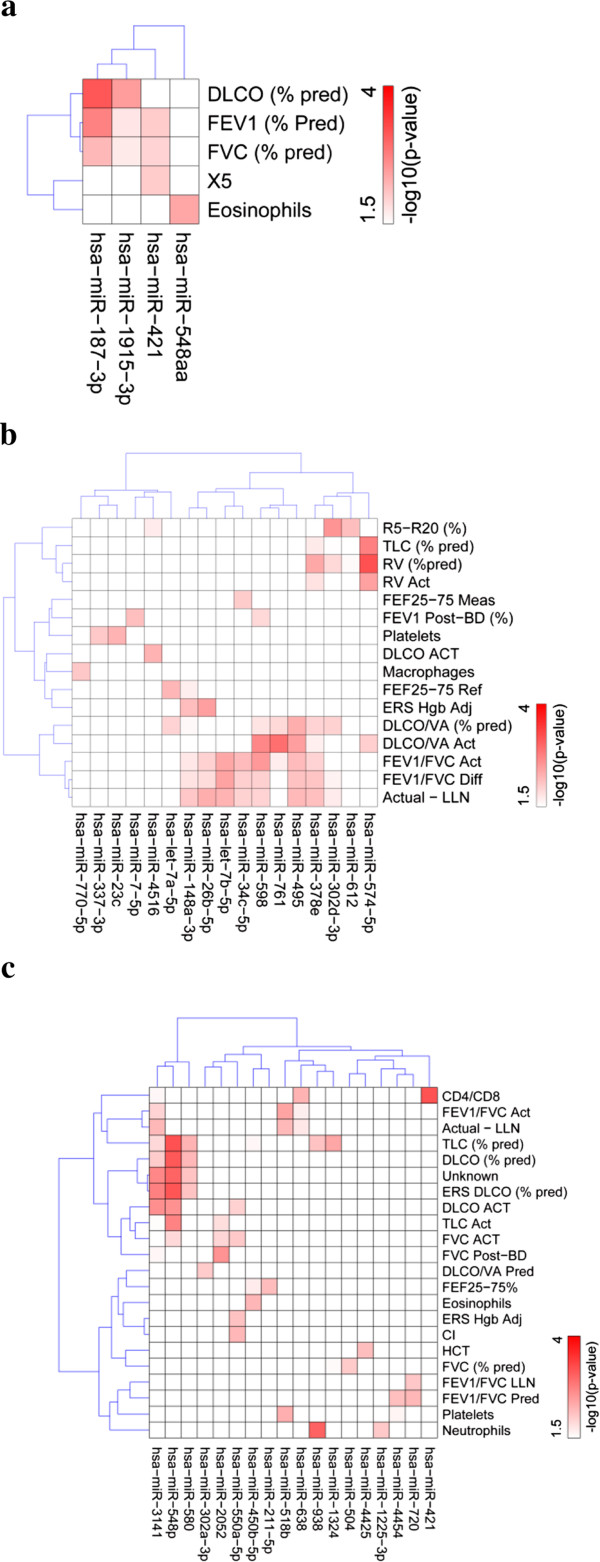
**Association of miRNA levels with clinical chart parameters.** Pearson correlations between different numerically continuous measurements of lung function or physiology and differentially expressed miRNAs were calculated for all STAMPEDE subjects. For simplicity, the negative log_10_ of calculated Pearson correlation values are displayed. Only relationships deemed to be potentially significant with *p*-value < 0.03 were depicted. **a**, BAL; **b**, Serum; **c**, Urine. DLCO, diffusing capacity for carbon monoxide; DLCO/VA, diffusing capacity for carbon monoxide adjusted for volume; ERS, European Respiratory Society; FEF25-75, mid-expiratory flow; FEV_1_, forced expiratory volume at one second; FVC, forced vital capacity; BD, bronchodilator; TLC, total lung capacity; RV, residual volume; IOS, impulse oscillometry; R5, total respiratory resistance; R20, proximal resistance; X5, distal capacitive reactance; Fres, resonant frequency; AX, reactance area; MCT, methacholine challenge testing; PFT, pulmonary function testing; HGB, hemoglobin; HCT, hematocrit; CD4/CD8, CD4 to CD8 lymphocyte ratio; WBC, white blood cell.

We calculated Pearson’s correlations between measured parameters of lung function and the large number of differentially expressed miRNAs that were detected in BAL, serum, or urine. To explore the relationship between them, a hierarchical clustering method was applied to the negative log10 of the Pearson’s correlation values as shown in Figure 7. In Figure 7a, miRNA 187-3p levels in STAMPEDE subjects shows possibly significant associations with diffusing capacity for carbon monoxide (DLCO) as well as forced expiratory volume at one second, percent of predicted (FEV1 (% pred)). This miRNA was also detected as differentially expressed between controls and STAMPEDEs in Figure 4. Several differentially expressed miRNAs that were identified in Figure 4, also had interesting correlations with clinical parameters: miRNAs 187-3p, 1915-3p, 4488, 421, and 371a-5p (data not shown). Associations between miRNA significantly expressed in serum with 15 clinical chart parameters are presented in Figure 7b and scatter plots of miRNA-574 against the data for residual volume (RV) and total lung capacity (TLC (% pred)) are given in Additional file 15. For urine, miRNAs 548p and 3141 showed potentially interesting associations with many related measures of lung function including TLC(% pred), DLCO (% pred), ERS DLCO (% pred) and DLCO ACT. Some of these are plotted in more detail in Additional file 13.

## Discussion

In this study we profiled proteins and miRNAs in clinical fluids to identify potential molecular markers for underlying lung disease in military personnel who deployed to Iraq or Afghanistan and were enrolled in the Army’s STAMPEDE study. The intent of this study was to identify proteins or miRNAs in soldiers who sought medical attention because of dyspnea within six months of redeployment. A retrospective review of veterans deployed to Southwest Asia suggested a higher rate of “new onset asthma” [31]. King et al., [32] reported a case series of 39 patients with constrictive bronchiolitis based on surgical lung biopsy; many of these patients also had an exposure to a sulfur mine fire. Overall the number of soldiers who self-reported with dyspnea to military treatment facilities remained low, given the large number of people who served in Iraq and Afghanistan (approximately 2.5 million) over the past ten years and the incidence of asthma in the adult US population of about 8% (http://www.cdc.gov/nchs/fastats/asthma.htm). For those soldiers with dyspnea, it is unclear whether this can be ascribed to solely to military service or whether it would have developed regardless. In part this was the rationale for the creation of the STAMPEDE project: to evaluate as many soldiers with dyspnea as possible at one location, develop diagnoses with conventional clinical tests, and use the patient registry for possible follow-up care. Samples of urine, serum and BAL were collected from a first cohort of STAMPEDE subjects. Since provisional diagnoses have been established for the STAMPEDE subjects, these diagnoses can be compared to the groups of differentially expressed proteins or the miRNAs that these subjects expressed. If one or more of the molecular profiles matches study subjects with the same conventional diagnoses, the molecular profiles become candidate biomarkers for that diagnosis. This would make it possible to supplement conventional lung disease diagnosis with a molecular profile. Such molecular profiles could become clinically useful biomarker profiles after additional validation studies. Highly specific and sensitive assays for serum-based protein biomarkers of lung cancer progression based on selective reaction monitoring (SRM) mass spectrometry are entering the clinic [33] and chip-based ELISA platforms have been described that can simultaneously measure multiple serum protein biomarkers from tiny samples of peripheral blood [34]. MiRNAs are potentially well-suited as disease biomarkers since they are present in virtually all body fluids [28] and have proven to be extremely stable in clinical samples of plasma, serum, urine, or BAL.

One group of six proteins was differentially expressed in the BAL and urine of STAMPEDE subjects compared to controls (Additional file 8) raising the possibility that these could have originated in the lung and passed into the urine. Further validation studies that could test for associations between these proteins and specific lung disorders could shift from accurate mass-tag or discovery proteomics methods to SRM mass spectrometry assays on small samples of BAL or urine. A second group of thirteen proteins that are typically found in plasma (AACT, A1AG1, A2MG) and others that are intracellular (HBA, HBB, CAH2: Table 1) were differentially expressed by a group of 10 STAMPEDE subjects (Figure 2). Five of these ten STAMPEDE subjects (21, 13, 15, 17, & 19) also showed the ‘group 1’ miRNA expression profile while three subjects (46, 36, 35) showed the second miRNA expression profile. Because of the overlap between these candidate marker profiles, these proteins should also be considered for validation studies. The finding of HBB and HBA which form adult hemoglobin in this group could suggest that lysed red blood cells were collected in the BAL. While localized bleeding could result from the bronchoscopy, it could also signal a disease process with some degree of tissue remodeling such as emphysema [35-37]. Follow-up studies could help choose between these possibilities.

We explored whether the two groups of STAMPEDE subjects that were defined by their miRNA profiles might have similar disease processes by comparing their preliminary diagnoses. The STAMPEDE subjects (11–18, 21, & 23) with the group 1 miRNA expression profile (Figure 4B) have provisional diagnoses of chronic obstructive pulmonary disease, fixed obstruction, airway hyperreactivity, air trapping, asthma, low DLCO (2 subjects), and constrictive bronchiolitis. We suggest that these diagnoses are consistent with a general finding of chronic airway disorders. The STAMPEDE subjects with the second miRNA expression profile (up-regulation of miR-191-5p, 71-5p, 125b-5p) had diagnoses of asthma (subjects 28 & 34), undiagnosed (subject 36), negative (subject 35), airway hyperreactivity (subject 43), abnormal cell count on BAL only (subject 44), or isolated low DLCO (subject 45). MiRNA 125b has been linked to inflammation [38]. In both groups of subjects having candidate biomarker signatures that are pointing to different stages of asthma-related lung disease, validation studies in which miRNA profiles are repeated and correlated with focused clinical tests become vitally important. Asthma may change over time: resolving in some individuals while progressing to more serious chronic conditions in others [39-41]. Based on the diagnoses for these subjects (Table 1) we speculate that it is entirely possible that the miRNA signature for group 2 subjects indicates a typical or early stage of asthma with active inflammation, while the signature for group 1 indicates a transitional condition which may have presented as asthma, but has now progressed through a phase of airway obstruction towards emphysema or COPD. Case/control or other validation studies could help clarify the value of the biomarkers we propose, by relating them to definite diagnoses.

Of the miRNAs that make up the group 1 profile, miR-371a-5p was strongly overexpressed in BAL and it was the only miRNA that was similarly differentially expressed in urine and serum as well BAL (Figure 6; Additional file 14). This miRNA is rare in blood [42] and it has not yet been linked to lung disorders. Thus identifying the cellular source(s) for this miRNA by *in situ* hybridization is an important follow-up goal, as well as correlating its expression with lung dysfunction. Mechanistically, miRNAs from the 371–373 family have been implicated as negative regulators of dickkopf 1 mRNA, which in turn permits activation of Wnt pathway signaling, which could lead to reactivation of cell growth or lung tissue remodeling [43]. MiRNA-421, also part of the group 1 signature was detected in urine as well as BAL. This miRNA has been proposed as a regulator of the DNA damage response, since it can be regulated by N-myc and it targets ATM, a key regulator of genome integrity and the DNA damage response [44,45]. Mir-421 has also been proposed as a regulator of pancreatic cancer [46] and as a biomarker for gastric cancer [47]. The other miRNAs that defined group 1 (1915-3p, 187-3p, 4488) have not been studied well and they have not been associated with lung disease before now. MiRNA 191, detected as part of the group 2 profile from lavage was also detected in serum. This miRNA was overexpressed in lung cancer [48] and it is rare in blood [42].

We also noticed that the levels of certain miRNAs correlated with clinical parameters of lung function. The Pearson’s correlation values were calculated between the set of all differentially expressed miRNAs and most numerically continuous lung clinic chart parameters [1]. Most of the miRNAs that constituted the signatures for group 1 and group 2 STAMPEDE subjects (Figure 4b) that were established by hierarchical cluster analysis were also detected by their extremely low Pearson’s correlation values with parameters such as DLCO or FEV (Figure 7). This increases our confidence that the associations of these miRNAs with pathologies that affect lung function, especially as measured by forced expiratory volume or gas exchange (DLCO) are meaningful. We recommend this approach for discovering new potential associations, particularly for generation of groups of miRNA candidates for subsequent validation studies. There is precedent for this approach since in another study, the levels of six miRNAs that were measured in induced sputum of smokers and patients with COPD showed correlations with FEV1 post (% pred) with r^2^ values ranging from 0.4 – 0.5 [49].

Protein and miRNA profiles from the control sample donors in this study is valuable because for the first time it establishes ‘wellness’ for normal or typical individuals, not known to have active lung disorders. Biomarkers of wellness as well as of diseases could be valuable in the future to judge lung health in routine physicals, return to normalcy after a lung procedure or disease, or a response to drug therapy for a lung condition such as asthma, fibrosis, or cancer. A panel of markers that evaluates lung health as well as the presence of common diseases could be especially valuable as part of the physical examination given at the time of recruitment, to identify problems or susceptibilities early, or before deployment to hazardous areas.

Another unexpected result was the finding that several miRNAs were expressed by most subjects with or without dyspnea. While some were expressed at about the same level in all subjects, the levels of others varied widely. We speculate that these may be derived from a fundamental lung cell or tissue such as bronchial smooth muscle or alveolar epithelium or alternatively from a cell that enters the lung from the circulation such as a macrophage, lymphocyte, or eosinophil. Departures from low- or baseline expression could be an indication of a disease or some other pathologic process. We are also investigating whether any of the differentially expressed miRNAs could be targeting the mRNAs for some of the differentially expressed proteins that were observed in this study.

Limitations on this work could include the fact that more than one underlying disease may be represented in the STAMPEDE subjects, since ‘dyspnea’ is a general description. We also profiled body fluids, not lung tissue itself and these profiles probably differ. Nonetheless some of the miRNAs that we detected have also been reported in studies of asthma or COPD. Two miRNAs we detected in serum (miR-223-3p and 15b-5p) we previously reported as being up-regulated in lung tissue from patients with COPD, compared with smokers with no lung obstruction [6]. We detected miR-449c-5p in both urine and serum of STAMPEDE subjects as well as miRs-449a and 34c-5p in urine. In two recent reports, the miRs from the miR-449/34 families were dysregulated in bronchial epithelial cells derived from patients with COPD [50] or asthma [51]. The miRNA expression pattern observed here from subjects with dyspnea, in particular the miRNAs that defined the group 1 subjects (371a-5p, 187-3p, 1915-3p, 4488, and 421) has not been reported yet for any lung disease. None of the miRNAs we detected as differentially expressed in lavage overlapped with a group of 17 miRNAs that has been proposed as a serum signature for non-small cell lung carcinoma [52,53]. We did detect one of these proposed markers (miR-1) in urine and another (miR-141-3p) in serum but there was no match to the complete signature for this type of lung cancer. Likewise, none of the miRs we reported overlapped with top-scoring pairs of miRNAs that were proposed as markers for three different pulmonary diseases (pneumonia, asthma, lung cancer) although miR-146*a*-5p, which we detected in serum differs by only 2 nucleotides from miR-146*b*-5p which had some diagnostic value in combination with other miRNAs [54]. Comparisons such as the above to previous work must be considered tentative because of the poor reproducibility between measurement platforms (see for example, [55]).

The STAMPEDE subjects, like most patients who experience breathing difficulty for the first time and come to the lung clinic for medical care do not know their diagnosis in advance. This was the motivation for the STAMPEDE study. Since dyspnea can be associated with diverse underlying conditions, it should be no surprise that the candidate markers we found were not clearly linked to one or two discrete clinical endpoints. But the patterns of markers we found were clearly separated from controls, and they fall into groups that could be associated with more or less advanced forms of asthma, based on our review of the available clinical findings. This work reveals that accurate lung disease diagnosis, even with excellent clinical support remains difficult, which underscores the potential value of objective molecular markers. The diagnostic value of the candidate markers we described can be judged in any future study where subjects have a well-defined lung disease.

## Conclusions

Profiling of proteins and miRNAs using advanced methods can give insights into the most detailed pathological as well as normal physiologic processes. The marker groups we have identified in soldiers with dyspnea may be closely related to lung disorders such as asthma, bronchiolitis, or COPD. As these correlations are made the candidate markers we described would be ready for validation studies and eventually, translation into clinical trials. The ultimate outcome would be novel platforms for new objective information to speed the reliable diagnosis of lung disorders.

## Abbreviations

FVC: Forced vital capacity; DLCO: Diffusing capacity lung, for carbon monoxide; BAL: Bronchoalveolar lavage fluid; miRNA: MicroRNA; DEG: Differentially expressed gene; DEmiRNA: Differentially expressed microRNA; (BCA): Bicinchoninic; (MS): Mass spectrometry; (AMT): Accurate mass and time; (FDR): False discovery rate; (DTT): Dithiothreitol.

## Competing interests

The authors declare that they have no competing interests. The views, opinions, and/or findings contained in this report are those of the authors and should not be construed as an official Department of the Army position, policy, or decision, unless so designated by other official documentation. Citations of commercial organizations or trade names in this report do not constitute an official Department of the Army endorsement or approval of the products or services of these organizations.

## Authors’ contributions

RG, MM, and JNB planned the experiments; AJS coordinated the clinical work; HMB, CDN, KKW, JA, RDS and JNB did the proteomics analysis; JHC and RG did the microRNA analysis; JNB, JA, MM and RG wrote the manuscript. All authors read and approved the manuscript.

## Pre-publication history

The pre-publication history for this paper can be accessed here:

http://www.biomedcentral.com/1755-8794/7/58/prepub

## Supplementary Material

Additional file 1 **Diagnoses and demographic data on STAMPEDE and control subjects.** See [1] for more details. Click here for file

Additional file 2 **Summary of correlation scores between datasets representing individuals.** Panels A, C, and E are of lung fluid proteomes and panels B, D, and F are urine proteomes. The 56 bars in panel A and the 60 bars in panel B represent the mean correlation (Pearson’s correlation coefficient, r) for a dataset compared to its biological replicates. The red horizontal line in panels A and B indicates the mean correlation threshold used to distinguish outliers, for lung fluid and urine, respectively. Outlier datasets are indicated by a red bar within the plots, while controls are green and disease are purple. Panels C and D are correlation heatmaps prior to outlier removal. The color of the cells in the heatmap correspond to the pairwise correlation coefficients between the row/column datasets, with red representing a perfect correlation (+1) and blue the minimal correlation value in the matrix. Panels E and F are the correlation heatmaps after outliers have been removed. The green and purple bars above and to the left of the correlation heatmaps designate control and disease, respectively. Click here for file

Additional file 3 **PCA plot of BAL and urine datasets on left and right, respectively.** Each dot represents an individual with green indicating control and purple designating disease individuals. Green and purple ellipses indicate the distribution of each group within the dimensions of the first and second principal components. Click here for file

Additional file 4 **Abundance and variance distributions for BAL and urine proteins.** (Upper left): normal quantile (Q-Q) plot of proteins identified from BAL fluid; (Upper right): variance distributions for BAL proteins from control subjects (green) or STAMPEDE subjects (violet); (Lower left): normal Q-Q plot of proteins identified from urine; (lower right): variance distributions for urine proteins from control subjects (green) or STAMPEDE subjects (violet). Click here for file

Additional file 5 **Analysis of technical replicates of miRNA levels.** The mean normalized counts are displayed along with the standard error of the mean. A: miRNA levels from two separate isolations from serum from subject 1 were profiled; B: miRNA from three separate isolations from urine from subject 1 were profiled; C: miRNA from two separate isolations from BAL from subject 2 were profiled. Click here for file

Additional file 6 **RT-PCR analysis of selected miRNA levels and comparison with NanoString levels.** MiRNAs that classified STAMPEDE subjects from controls (187-3p, 371a-5p, 212-3p, 1915-3p) and miRNAs that were strongly expressed in BAL but did not discriminate STAMPEDE subjects from controls (4516, 320e, and 630) were measured by quantitative RT-PCR as described in Methods. RT-PCR amplifications were run in quadruplicate for all samples and the results were averaged. The average expression of these miRNAs as observed in STAMPEDE subjects 12–14 & 16–18 and in control subjects 1, 4–7, & 12 are compared. STAMPEDE subjects 12–14 and 16–18 were classified together as belonging to a sub-group that shared a similar profile of miRNA expression which was quite distinct from the controls. A: Normalized levels of the indicated miRNAs in STAMPEDE subjects (S-187, S-371, etc.) or controls (C-187, C-371, etc.) by NanoString profiling. The standard error of the mean is shown. B: Levels of the indicated miRNAs in subjects or controls by RT-PCR. The standard error of the mean is shown. Click here for file

Additional file 7 **Heatmap of the 79 significantly different proteins in lung fluid between control and disease individuals, designated by the light and dark blue bars above the heatmap, respectively.** Protein abundance values were clustered by unsupervised hierarchical clustering using Pearson’s correlation as a measure of distance and complete agglomeration, which is default for the R ‘hclust’ method. The protein abundance values were scaled using z-scores, with red representing 1 standard deviation above the mean and green being 1 standard deviation below the mean. Uniprot accession identifiers for the proteins are shown on the right side of the heatmap. Click here for file

Additional file 8 **Proteins reliably identified in common from BAL (n = 79) and urine (n = 74) from Additional files**3**&**4** are listed by their UniProt accession identifiers.** The proteins in common from BAL and urine are listed as well. Click here for file

Additional file 9 **A, Gene ontogeny terms (GO) for BAL or urine proteins.** Twenty-eight of seventy-nine proteins were used for this search. B: Gene ontogeny terms (GO) for urine proteins. Thirty-seven of seventy-four proteins were used for this analysis. Click here for file

Additional file 10 **Heatmap of the 74 significantly different proteins in urine between control and disease individuals, designated by the light and dark blue bars above the heatmap, respectively.** The protein abundance values were scaled using z-score, with red representing 1 standard deviation above the mean and green being 1 standard deviation below the mean. Uniprot accession identifiers for the proteins are shown on the right side of the heatmap. Click here for file

Additional file 11 **Box plots for the levels of miR-187-3p, 371a-5p, 421, 1915-3p, and 4488 define STAMPEDE subjects in group 1 compared with controls.** Group 1: STAMPEDE subjects 16, 18, 13, 17, 15, 21, 12, 23, 14, & 11; Control: all 15 control subjects; Control + others: controls plus STAMPEDE subjects exclusive of group 1. Click here for file

Additional file 12 One way cluster analysis of 76 differentially expressed miRNAs in urine. Click here for file

Additional file 13 One way cluster analysis of 96 differentially expressed miRNAs in serum. Click here for file

Additional file 14 **Pair-wise expression of miRNA in BAL, urine, or serum.** Upper left, the average expression of a specific miRNA (as the log_2_ level) in urine plotted as a function of its average levels in BAL; Upper right, miRNA expression in serum as a function of the levels in BAL; Lower left, miRNA expression in urine as a function of the levels in serum. The blue arrow identifies the expression of miRNA 371a-5p which was elevated in all three samples from STAMPEDE subjects. While the majority of the miRNA epression changes in BAL, urine, and serum were independent of one another, miRNA 371a-5p was consistently overexpressed in BAL, urine, and serum from STAMPEDE subjects. Click here for file

Additional file 15 Plots of selected miRNAs that showed associations with medical chart parameters by calculation of Pearson correlations. Click here for file

## References

[B1] Morris M Dodson D Lucero P Haislip G Gallup R Nicholson K Zacher L Study of active duty military for pulmonary disease related to environmental deployment exposures (STAMPEDE) Am J Respir Crit Care Med 2014 190 77 84 10.1164/rccm.201402-0372OC 24922562

[B2] Maisel A Mueller C Nowak R Peacock W Landsberg J Ponikowski P Mockel M Hogan C Wu A Richards M Clopton P Filippatos G Di Somma S Anand I Ng L Daniels L Neath S Christenson R Potocki M McCord J Terracciano G Kremastinos D Hartmann O von Haehling S Bergmann A Morgenthaler N Anker S Mid-region Pro-hormone markers for diagnosis and prognosis in acute DyspneaResults from the BACH (biomarkers in acute heart failure) trial J Am Coll Cardiol 2010 55 2062 2076 10.1016/j.jacc.2010.02.025 20447528

[B3] Szefler S Wenzel S Brown R Erzurum S Fahy J Hamilton R Hunt J Kita H Liu A Panettieri R Schleimer R Minnicozzi M Asthma outcomes: biomarkers J Allergy Clin Immunol 2012 129 S9 S23 10.1016/j.jaci.2011.12.979 22386512 PMC3390196

[B4] Erzurum S Gaston B Biomarkers in asthma: a real hope to better manage asthma Clin Chest Med 2012 33 459 471 10.1016/j.ccm.2012.06.007 22929095 PMC3494969

[B5] Sircar G Saha B Bhattacharya S Saha S Allergic asthma biomarkers using systems approaches Front Genet 2013 4 308 doi:10.3389/fgene.2013.00308; PMCID: PMC3884215 24409194 10.3389/fgene.2013.00308 PMC3884215

[B6] Ezzie M Crawford M Cho J Orellana R Zhang S Gelinas R Batte K Yu L Nuovo G Galas D Diaz P Wang K Nana-Sinkam P Gene expression networks in COPD: microRNA and mRNA regulation Thorax 2011 doi:10.1136/thoraxjnl-2011-200089 10.1136/thoraxjnl-2011-200089 21940491

[B7] Pinto-Plata V Mullerova H Casanova C de Torres J Corado H Varo N Cordoba E Baz R Cortopasi F Divo M Kelly E Celli B Serum biomarkers in COPD survivors and controls. Longitudinal analysis Am J Respir Crit Care Med 2012 185 A4507

[B8] Cho J Gelinas R Wang K Etheridge A Piper M Batte K Dakhlallah D Price J Bornman D Zhang S Marsh C Galas D Systems biology of interstitial lung diseases: integration of mRNA and microRNA expression changes BMC Med Genet 2011 4 8 10.1186/1755-8794-4-8 PMC3035594 21241464

[B9] Park M Vittinghoff E Liu K Shlipak M Hsu C Urine biomarkers neutrophil gelatinase-associated lipocalin (NGAL) and kidney injury molecule-1 (KIM-1) have different patterns in heart failure exacerbation Biomark Insights 2013 8 15 23531625 10.4137/BMI.S11479 PMC3603387

[B10] Parikh C Butrymowicz I Yu A Chinchilli V Park M Hsu C Reeves W Devarajan P Kimmel P Siew E Liu K Urine stability studies for novel biomarkers of acute kidney injury Am J Kidney Dis 2014 63 567 572 10.1053/j.ajkd.2013.09.013 24200462 PMC3969397

[B11] Roobol MJ Haese A Bjartell A Tumour markers in prostate cancer III: biomarkers in urine Acta Oncol 2011 50 85 89 10.3109/0284186X.2010.524935 21604945

[B12] Ploussard G de la Taille A Urine biomarkers in prostate cancer Nat Rev Urol 2010 7 101 109 10.1038/nrurol.2009.261 20065953

[B13] Ben-Dov I Tan Y Morozov P Wilson P Rennert H Blumenfeld J Tuschl T Urine MicroRNA as potential biomarkers of autosomal dominant polycystic kidney disease progression: description of miRNA profiles at baseline PLoS One 2014 9 e86856 10.1371/journal.pone.0086856 24489795 PMC3906110

[B14] Maiolica A Borsotti D Rappsilber J Self-made frits for nanoscale columns in proteomics Proteomics 2005 5 3847 3850 10.1002/pmic.200402010 16130174

[B15] Kelly RT Page JS Luo Q Moore RJ Orton DJ Tang K Smith R Chemically etched open tubular and monolithic emitters for nanoelectrospray ionization mass spectrometry Anal Chem 2006 78 7796 7801 10.1021/ac061133r 17105173 PMC1769309

[B16] Zimmer JS Monroe ME Qian WJ Smith R Advances in proteomics data analysis and display using an accurate mass and time tag approach Mass Spectrom Rev 2006 25 450 482 10.1002/mas.20071 16429408 PMC1829209

[B17] Stanley JR Adkins JN Slysz GW Monroe ME Purvine SO Karpievitch YV Anderson GA Smith RD Dabney AR A statistical method for assessing Peptide identification confidence in accurate mass and time tag proteomics Anal Chem 2011 83 6135 6140 10.1021/ac2009806 21692516 PMC3212438

[B18] Jaitly N Mayampurath A Littlefield K Adkins JN Anderson GA Smith RD Decon2LS: an open-source software package for automated processing and visualization of high resolution mass spectrometry data BMC Bioinform 2009 10 87 doi:10.1186/1471-2105-10-87 10.1186/1471-2105-10-87 PMC2666663 19292916

[B19] Monroe ME Tolić N Jaitly N Shaw JL Adkins JN Smith RD VIPER: an advanced software package to support high-throughput LC-MS peptide identification Bioinformatics 2007 23 2021 2023 Epub 2007 Jun 1 10.1093/bioinformatics/btm281 17545182

[B20] Webb-Robertson BJ Matzke MM Jacobs JM Pounds JG Waters KM A statistical selection strategy for normalization procedures in LC-MS proteomics experiments through dataset-dependent ranking of normalization scaling factors Proteomics 2011 11 4736 4741 10.1002/pmic.201100078 22038874 PMC3517140

[B21] Norbeck A Monroe M Adkins J Smith R The utility of accurate mass and LC elution time information in the analysis of complex proteomes J Am Soc Mass Spectrom 2005 16 1239 1249 10.1016/j.jasms.2005.05.009 15979333 PMC1769320

[B22] Matzke MM Waters KM Metz TO Jacobs JM Sims AC Baric RS Pounds JG Webb-Robertson BJ Improved quality control processing of peptide-centric LC-MS proteomics data Bioinformatics 2011 27 2866 2872 10.1093/bioinformatics/btr479 21852304 PMC3187650

[B23] Huang D Sherman B Tan Q Kir J Liu D Bryant D Guo Y Stephens R Baseler M Lane H Lempicki R DAVID bioinformatics resources: expanded annotation database and novel algorithms to better extract biology from large gene lists Nucleic Acids Res 2007 35 suppl 2 W169 W175 17576678 10.1093/nar/gkm415 PMC1933169

[B24] Clough T Key M Ott I Ragg S Schadow G Vitek O Protein quantification in label-free LC-MS experiments J Proteome Res 2009 8 5275 5284 10.1021/pr900610q 19891509

[B25] Benjamini Y Hochberg Y Controlling the false discovery rate - a practical and powerful approach to multiple testingj Roy Stat Soc B Met 1995 57 289 300

[B26] Team RDC R: A Language and Environment for Statistical Computing 2008 Vienna, Austria: R Foundation for Statistical Computing

[B27] Geiss G Bumgarner R Birditt B Dahl T Dowidar N Dunaway D Fell H Ferree S George R Grogan T James J Maysuria M Mitton J Oliveri P Osborn J Peng T Ratcliffe A Webster P Davidson E Hood L Krassen D Direct multiplexed measurement of gene expression with color-coded probe pairs Nat Biotechnol 2008 26 317 325 10.1038/nbt1385 18278033

[B28] Weber J Baxter D Zhang S Huang D Huang K Lee M Galas D Wang K The microRNA spectrum in 12 body fluids Clin Chem 2010 2010 56 1733 1741 doi:10.1373/clinchem.2010.147405 20847327 10.1373/clinchem.2010.147405 PMC4846276

[B29] Waggott D Chu K Yin S Wouters B Liu F Boutros P NanoStringNorm Bioinformatics 2012 28 1546 1548 10.1093/bioinformatics/bts188 22513995 PMC3356845

[B30] Smyth GK Linear models and empirical Bayes methods for assessing differential expression in microarray experiments Stat Appl Genet Mol Biol 2004 3 1 Article 3 10.2202/1544-6115.1027 16646809

[B31] Szema AM Peters MC Weissinger KM Gagliano CA Chen J New-onset asthma among soldiers serving in Iraq and Afghanistan Allergy Asthma Proc 2010 31 67 71 doi:10.2500/aap.2010.31.3383 20929596 10.2500/aap.2010.31.3383

[B32] King M Eisenberg R Newman J Tolle J Harrell F Nian H Ninan M Lambright E Sheller J Johnson J Miller R Constrictive bronchiolitis in soldiers returing from Iraq and Afghanistan N Engl J Med 2011 365 222 230 doi:10.1056/NEJMoa1101388 10.1056/NEJMoa1101388 21774710 PMC3296566

[B33] Li XJ Hayward C Fong PY Dominguez M Hunsucker SW Lee LW McLean M Law S Butler H Schirm M Gingras O Lamontagne J Allard R Chelsky D Price ND Lam S Massion PP Pass H Rom WN Vachani A Fang KC Hood L Kearney P A blood-based proteomic classifier for the molecular characterization of pulmonary nodules Sci Transl Med 2013 5 207ra 142 10.1126/scitranslmed.3007013 PMC4114963 24132637

[B34] Fan R Vermesh O Srivastava A Yen B Qin L Ahmad H Kwong G Liu C Gould J Hood L Heath J Integrated barcode chips for rapid, multiplexed analysis of proteins in microliter quantities of blood Nat Biotechnol 2008 26 1373 1378 doi:10.1038/nbt.1507 10.1038/nbt.1507 19029914 PMC2775523

[B35] Dorfmüller P Pulmonary Hypertension: Pathology Pharmacotherapy of Pulmonary Hypertension 2013 Heidelberg: Springer Berlin 59 75

[B36] de Prost N Parrot A Cuquemelle E Picard C Cadranel J Immune diffuse alveolar hemorrhage: a retrospective assessment of a diagnostic scale Lung 2013 191 559 563 10.1007/s00408-013-9491-3 23867964

[B37] Puxeddu E Comandini A Cavalli F Pezzuto G D’Ambrosio C Senis L Paci M Curradi G Sergiacomi G Saltini C Iron laden macrophages in idiopathic pulmonary fibrosis: the telltale of occult alveolar hemorrhage? Pulmonary Pharmacol Therapeutics 2014 28 35 40 10.1016/j.pupt.2013.12.002 24365112

[B38] Huang H Yu H Huang L Huang HC Chen R Lin I Ou C Hsu T Yang K miRNA-125b regulates TNF-α production in CD14+ neonatal monocytes via post-transcriptional regulation J Leukoc Biol 2012 92 171 182 10.1189/jlb.1211593 22581933

[B39] Cohn L Elias J Chupp G Asthma: mechanisms of disease persistence and progression Annu Rev Immunol 2004 22 789 815 10.1146/annurev.immunol.22.012703.104716 15032597

[B40] Broide DH Immunologic and inflammatory mechanisms that drive asthma progression to remodeling J Allergy Clin Immunol 2008 121 560 570 10.1016/j.jaci.2008.01.031 18328887 PMC2386668

[B41] Royce S Cheng V Samuel C Tang M The regulation of fibrosis in airway remodeling in asthma Mol Cell Endocrinol 2012 351 167 175 10.1016/j.mce.2012.01.007 22266540

[B42] Pritchard C Kroh E Wood B Arroyo J Dougherty K Miyaji M Tait J Tewari M Blood cell origin of circulating microRNAs: a cautionary note for cancer biomarker studies Cancer Prevention Res 2012 5 492 497 doi:10.1158/1940-6207.capr-11-0370 10.1158/1940-6207.CAPR-11-0370 PMC4186243 22158052

[B43] Zhou A Diao L Xu H Xiao Z Li J Xhou H Qu L β-catenin/LEF1 transactivates the microRNA-371-373 cluster that modulates the Wnt/β-catenin pathway Oncogene 2012 31 2968 2978 10.1038/onc.2011.461 22020335

[B44] Hu H Du L Nagabayashi G Seeger R Gatti R ATM is down-regulated by N-Myc-regulated microRNA-421 Proc Natl Acad Sci U S A 2010 107 1506 1511 10.1073/pnas.0907763107 20080624 PMC2824372

[B45] Hu H Gatti R MicroRNAs: new players in the DNA damage response J Mol Cell Biol 2011 3 151 158 10.1093/jmcb/mjq042 21183529 PMC3104011

[B46] Hao J Zhang S Zhou Y Liu C Hu X Shao C MicroRNA 421 suppresses *DPC4/Smad4* in pancreatic cancer Biochem Biophys Res Comm 2011 406 552 557 10.1016/j.bbrc.2011.02.086 21352803

[B47] Zhang X Cui L Ye G Zheng T Song H Xia T Yu X Xiao B Le Y Guo J Gastric juice microRNA-421 is a new biomarker for screening gastric cancer Tumor Biol 2012 33 2349 2355 10.1007/s13277-012-0497-x 22926798

[B48] Yanaihara N Caplen N Bowman E Seike M Kumamoto K Yi M Stephens R Okamoto A Yokota J Tanaka T Calin G Liu C Croce C Harris C Unique microRNA molecular profiles in lung cancer diagnosis and prognosis Cancer Cell 2006 9 189 198 10.1016/j.ccr.2006.01.025 16530703

[B49] Van Pottelberge G Mestdagh P Bracke K Thas O van Durme Y Joos G Vandesompele J Brusselle G MicroRNA expression in induced sputum of smokers and patients with chronic obstructive pulmonary disease Am J Respir Crit Care Med 2011 183 898 906 10.1164/rccm.201002-0304OC 21037022

[B50] Wang T Campbell J Liu G LeClerc A Alekseyev Y Luo L Xiao J Zhang X Sin D McWilliams A Lam S Spira A Lenburg M Bronchial airway microRNA expression associated with chronic obstructive pulmonary disease Am J Respir Crit Care Med 2013 187 A1201

[B51] Solberg O Ostrin E Love M Peng J Bhakta N Hou L Nguyen C Solon M Nguyen C Barczak A Zlock L Blagev D Finkbeiner W Ansel K Arron J Erle D Woodruff P Airway epithelial miRNA expression is altered in asthma Am J Respir Crit Care Med 2012 186 965 974 doi:10.1164/rccm.201201-00170C 10.1164/rccm.201201-0027OC 22955319 PMC3530212

[B52] Wang J Zhang K-Y Liu S-M Sen S Tumor-associated circulating microRNAs as biomarkers of cancer Molecules 2014 19 1912 1938 doi:10.3390/molecules19021912 10.3390/molecules19021912 24518808 PMC6271223

[B53] Boeri M Verri C Conte D Roz L Modena P Facchinetti F Calabro E Croce C Pastorino U Sozzi G MicroRNA signatures in tissues and plasma predict development and prognosis of computed tomography detected lung cancer Proc Natl Acad Sci U S A 2011 108 3713 3718 doi/10.1073/pnas.1100048108 10.1073/pnas.1100048108 21300873 PMC3048155

[B54] Sheinerman K Tsivinsky V Umansky S Analysis of organ-enriched microRNAs in plasma as an approach to development of universal screening test: feasibility study J Transl Med 2013 11 304 doi:10.1186/1479-5876-11-304 10.1186/1479-5876-11-304 24330742 PMC3867418

[B55] Sah S McCall M Eveleigh D Wilson M Irizarry R Performance evaluation of commercial miRNA expression array platforms BMC Res Notes 2010 3 80 doi:10.1186/1756-0500-3-80 10.1186/1756-0500-3-80 20298588 PMC2853548

