# Supplementary material for: Protein and microRNA biomarkers from lavage, urine, and serum in military personnel evaluated for dyspnea
Source: BMC Med Genomics. 2014 Oct 5;7:58. doi: 10.1186/1755-8794-7-58 (PMC4193960; doi:10.1186/1755-8794-7-58)
Supplement: Additional file 1 — Diagnoses and demographic data on STAMPEDE and control subjects. See [1] for more details. [file 1755-8794-7-58-S1.docx]

| STAMPEDE or CONTROL |  |  |  |  |
| --- | --- | --- | --- | --- |
|  |  |  |  |  |
|  | **Final Diagnosis** | **Gender** | **Race** | **Age** |
|  |  |  |  |  |
| S1 | Undiagnosed | M | C | 32 |
| S2 | Undiagnosed | M | C | 24 |
| S3 | Undiagnosed | M | H | 27 |
| S4 | Undiagnosed | M | C | 24 |
| S5 | AHR | M | C | 26 |
| S6 | Asthma | M | H | 41 |
| S7 | Undiagnosed | M | H | 34 |
| S8 | Asthma | M | C | 21 |
| S9 | Undiagnosed | F | B | 40 |
| S10 | AHR | M | C | 21 |
| S11 | Undiagnosed | M | C | 21 |
| S12 | Obstruction | M | C | 23 |
| S13 | Low DLCO | M | C | 29 |
| S14 | Air Trapping | M | H | 34 |
| S15 | Constrictive bronchiolitis | n.a. | n.a | n.a |
| S16 | Low DLCO | F | B | 28 |
| S17 | Asthma | M | C | 39 |
| S18 | Undiagnosed | M | C | 24 |
| S19 | AHR | M | H | 22 |
| S20 | Abnormal CC | M | C | 38 |
| S21 | AHR | M | H | 28 |
| S22 | Asthma | M | C | 22 |
| S23 | COPD | M | C | 40 |
| S24 | Abnormal CC | M | H | 46 |
| S25 | Abnormal CC | M | C | 37 |
| S26 | AHR | F | H | 30 |
| S27 | AHR | M | C | 23 |
| S28 | Asthma | F | C | 37 |
| S29 | Lung Nodule | F | B | 39 |
| S30 | Abnormal CC | M | C | 51 |
| S31 | Undiagnosed | M | B | 45 |
| S32 | low DLCO | n.a. | n.a | n.a |
| S33 | Inhalation Injury | M | C | 31 |
| S34 | Asthma | M | C | 31 |
| S35 | Negative | n.a. | n.a | n.a |
| S36 | Undiagnosed | M | C | 26 |
| S37 | Abnormal CC | M | H | 28 |
| S38 | Asthma | F | B | 29 |
| S39 | Asthma | n.a. | n.a | n.a |
| S40 | Asthma/VCD | F | C | 30 |
| S41 | Undiagnosed | M | C | 32 |
| S42 | dynamic airway collapse | n.a. | n.a | n.a |
| S43 | GERD/AHR | M | C | 41 |
| S44 | Abnormal CC | M | B | 46 |
| S45 | Low DLCO | M | C | 23 |
| S46 | Undiagnosed | M | H | 23 |
| S47 | AHR | M | B | 37 |
| S48 | Undiagnosed | F | B | 41 |
| C1 | control | M | H | 29 |
| C2 | control | M | W | 28 |
| C3 | control | F | W | 28 |
| C4 | control | M | W | 30 |
| C5 | control | M | W | 32 |
| C6 | control | M | W | 28 |
| C7 | control | F | W | 29 |
| C8 | control | M | W | 30 |
| C9 | control | M | W | 31 |
| C10 | control | F | H | 26 |
| C11 | control | F | W | 27 |
| C12 | control | M | W | 27 |
| C13 | control | M | W | 30 |
| C14 | control | M | H | 33 |
| C15 | control | F | W | 28 |

Legend: S1, STAMPEDE subject 1; C1, control subject 1; Gender, STAMPEDE M, 35 (80%), F, 8 (20%), control M, 10(67%), F, 5 (33%); Race, B—Black; H—Hispanic; W—White; STAMPEDE, W, 25(58%), H 10(23%), B, 8(19%); Control, W, 12 (80%), H, 3 (20%); n.a, not available.
