# Supplementary figures and images for: Protein and microRNA biomarkers from lavage, urine, and serum in military personnel evaluated for dyspnea
Source: BMC Med Genomics. 2014 Oct 5;7:58. doi: 10.1186/1755-8794-7-58 (PMC4193960; doi:10.1186/1755-8794-7-58)

**
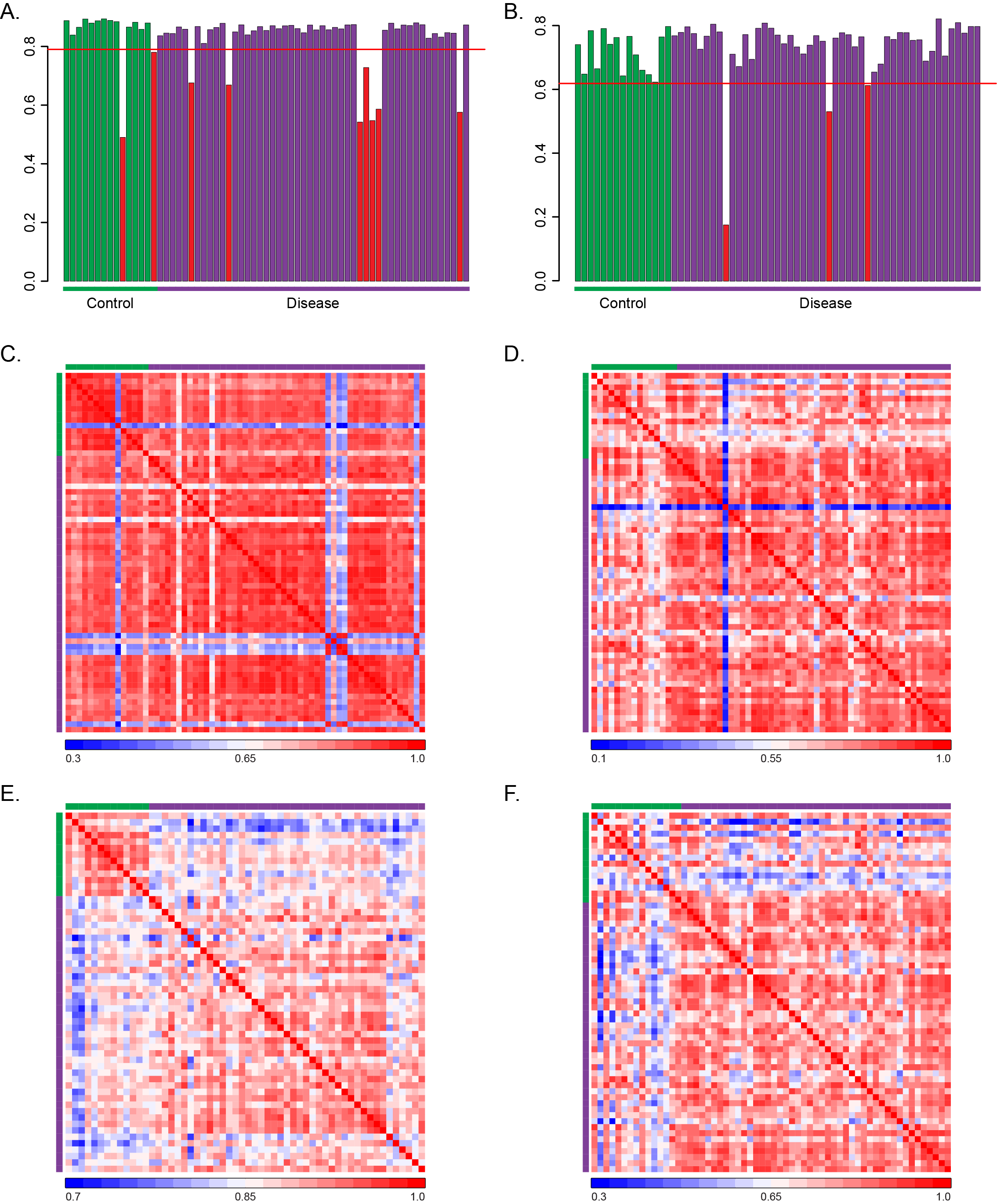
**

Supplement: Additional file 2 — Summary of correlation scores between datasets representing individuals. Panels A, C, and E are of lung fluid proteomes and panels B, D, and F are urine proteomes. The 56 bars in panel A and the 60 bars in panel B represent the mean correlation (Pearson’s correlation coefficient, r) for a dataset compared to its biological replicates. The red horizontal line in panels A and B indicates the mean correlation threshold used to distinguish outliers, for lung fluid and urine, respectively. Outlier datasets are indicated by a red bar within the plots, while controls are green and disease are purple. Panels C and D are correlation heatmaps prior to outlier removal. The color of the cells in the heatmap correspond to the pairwise correlation coefficients between the row/column datasets, with red representing a perfect correlation (+1) and blue the minimal correlation value in the matrix. Panels E and F are the correlation heatmaps after outliers have been removed. The green and purple bars above and to the left of the correlation heatmaps designate control and disease, respectively. [file 1755-8794-7-58-S2.docx]

**
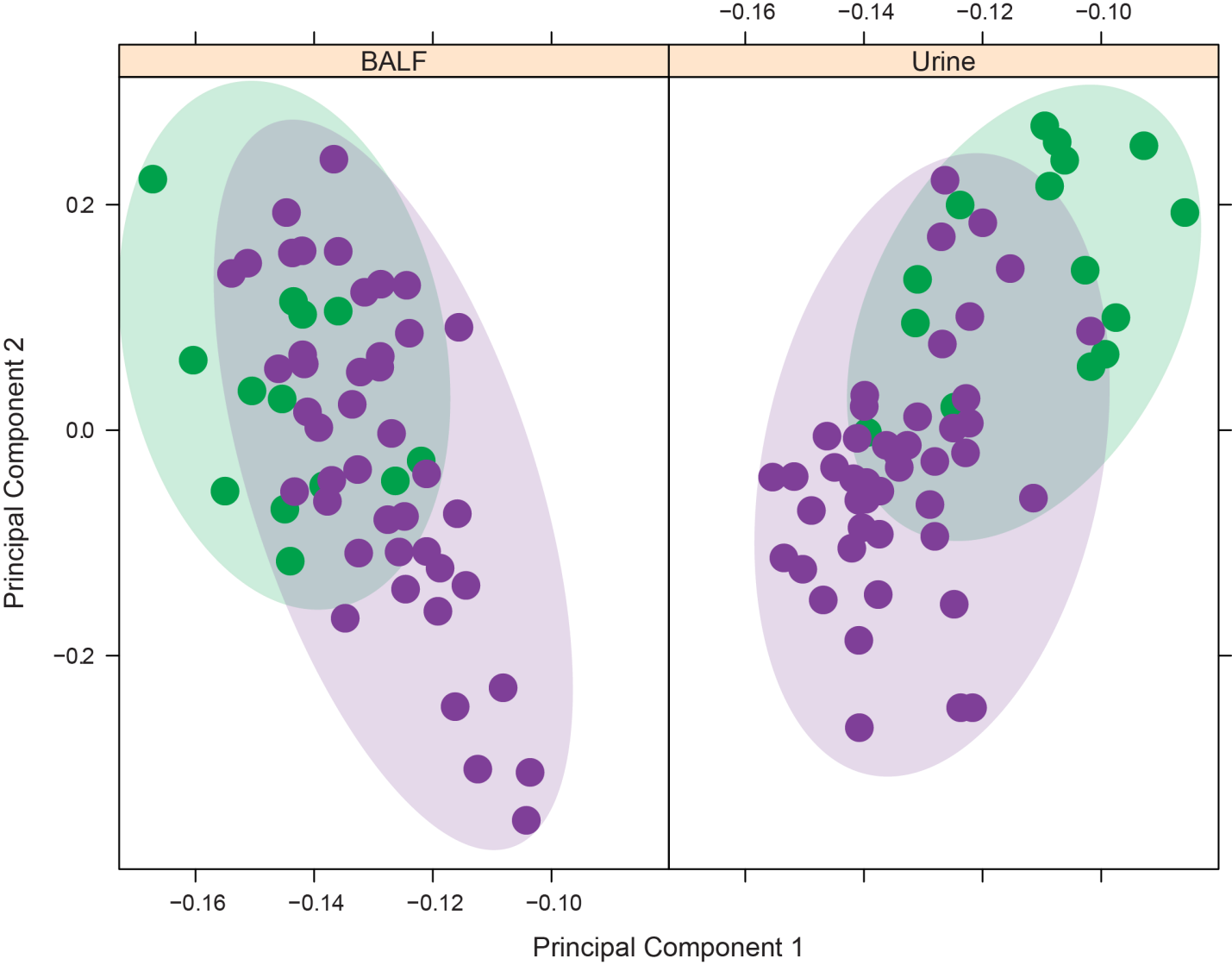
**

Supplement: Additional file 3 — PCA plot of BAL and urine datasets on left and right, respectively. Each dot represents an individual with green indicating control and purple designating disease individuals. Green and purple ellipses indicate the distribution of each group within the dimensions of the first and second principal components. [file 1755-8794-7-58-S3.docx]

**
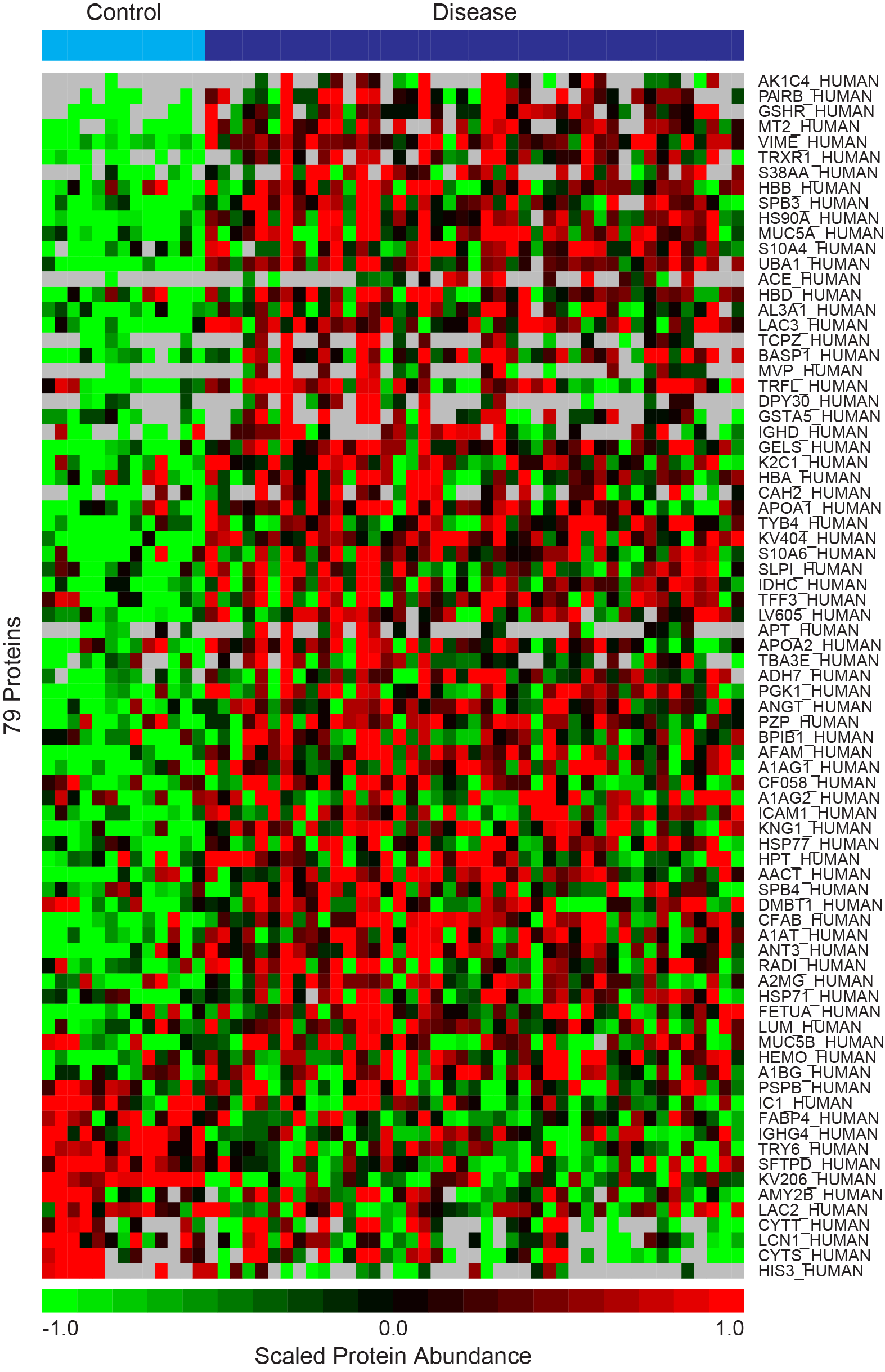
**

Patients

Supplement: Additional file 7 — Heatmap of the 79 significantly different proteins in lung fluid between control and disease individuals, designated by the light and dark blue bars above the heatmap, respectively. Protein abundance values were clustered by unsupervised hierarchical clustering using Pearson’s correlation as a measure of distance and complete agglomeration, which is default for the R ‘hclust’ method. The protein abundance values were scaled using z-scores, with red representing 1 standard deviation above the mean and green being 1 standard deviation below the mean. Uniprot accession identifiers for the proteins are shown on the right side of the heatmap. [file 1755-8794-7-58-S7.docx]

Additional file 9.

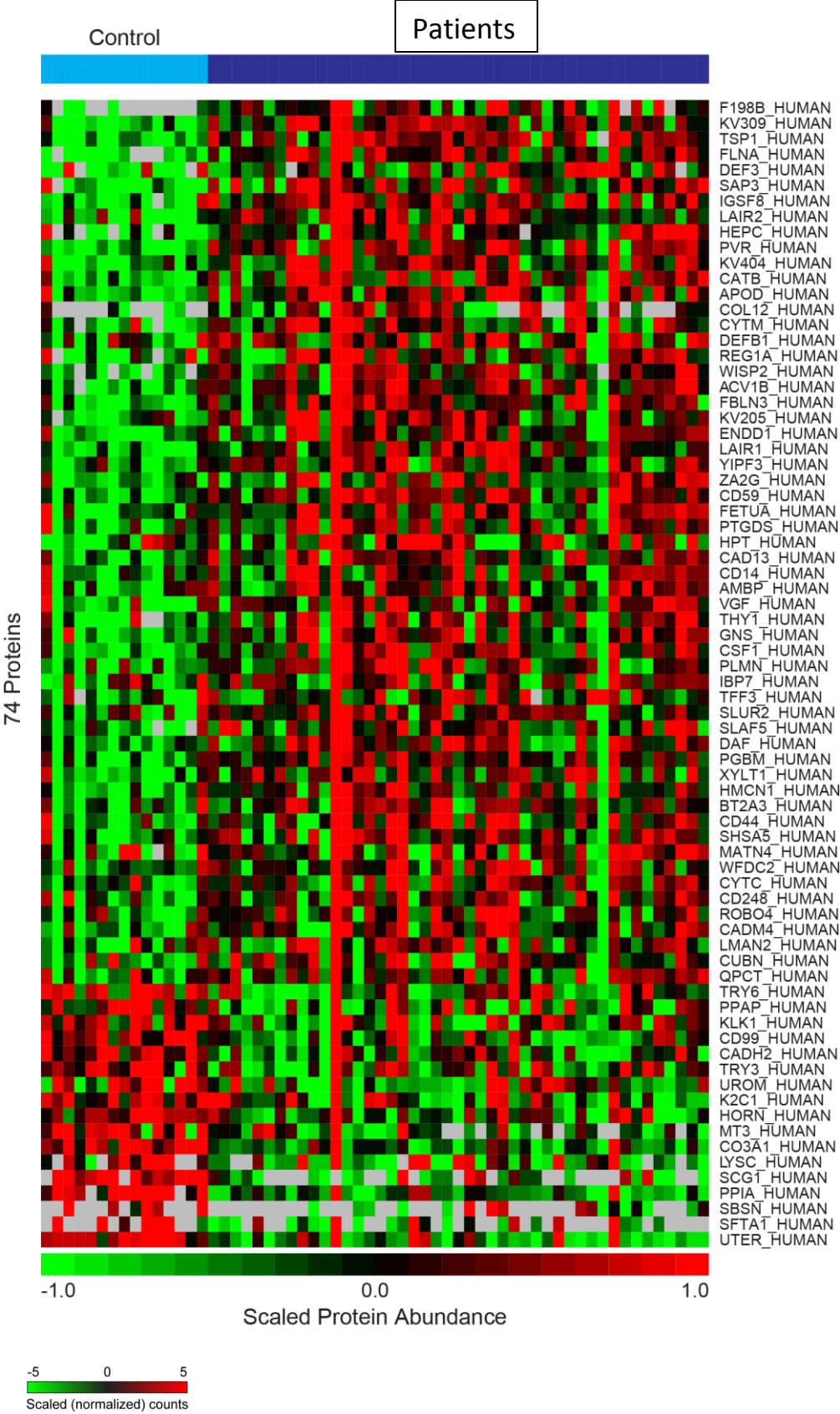

Supplement: Additional file 9 — A, Gene ontogeny terms (GO) for BAL or urine proteins. Twenty-eight of seventy-nine proteins were used for this search. B: Gene ontogeny terms (GO) for urine proteins. Thirty-seven of seventy-four proteins were used for this analysis. [file 1755-8794-7-58-S9.pdf]

Patients

**
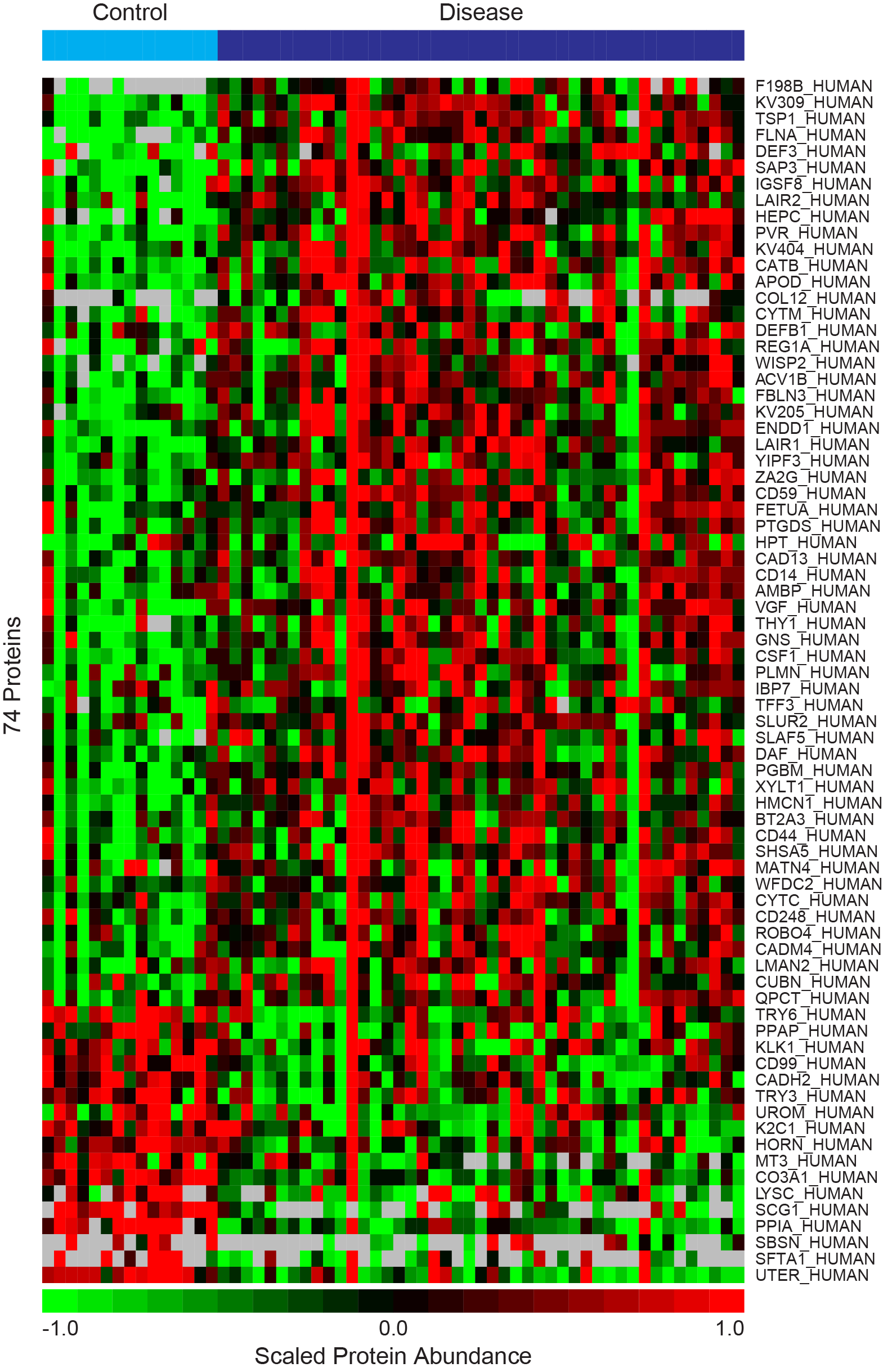
**


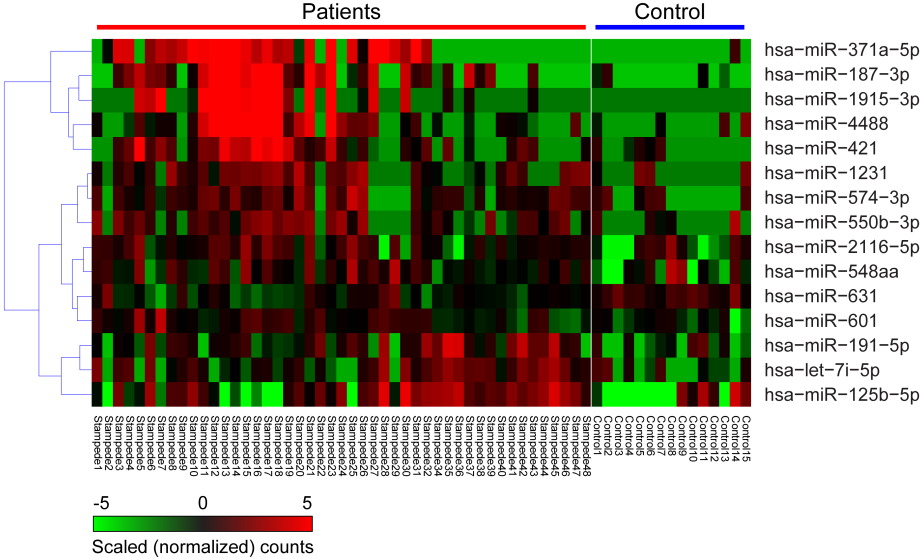

Supplement: Additional file 10 — Heatmap of the 74 significantly different proteins in urine between control and disease individuals, designated by the light and dark blue bars above the heatmap, respectively. The protein abundance values were scaled using z-score, with red representing 1 standard deviation above the mean and green being 1 standard deviation below the mean. Uniprot accession identifiers for the proteins are shown on the right side of the heatmap. [file 1755-8794-7-58-S10.docx]

hsa-miR-187-3p

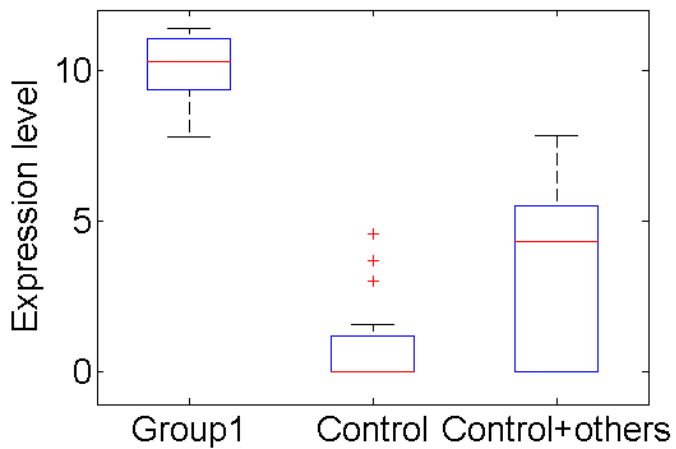

hsa-miR-371a-5p

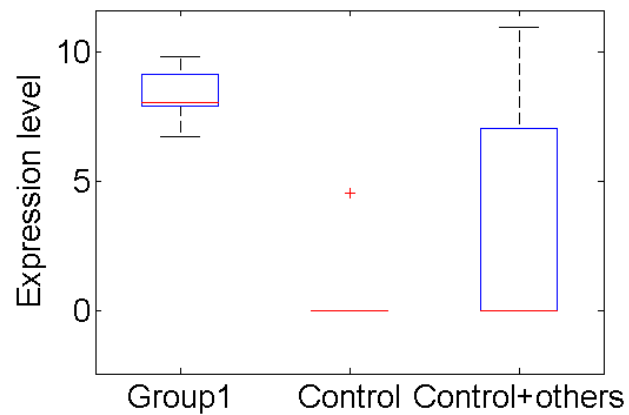

hsa-miR-421

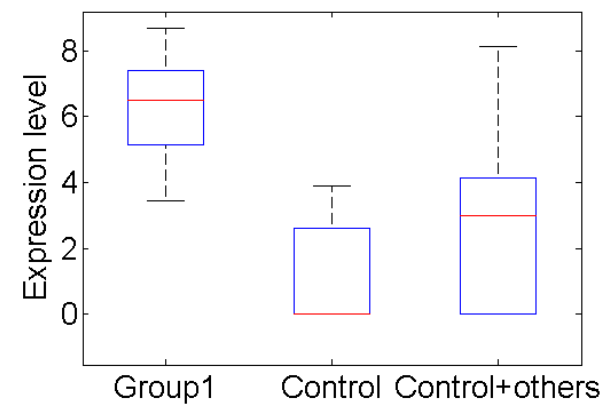

hsa-miR-1915-3p

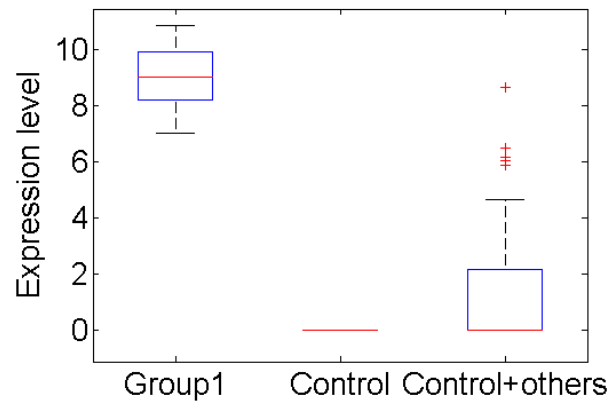

hsa-miR-4488

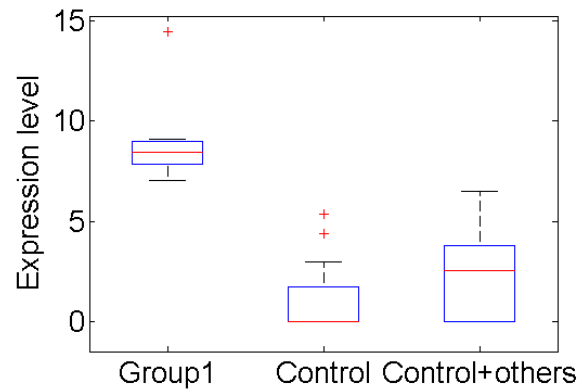

Supplement: Additional file 11 — Box plots for the levels of miR-187-3p, 371a-5p, 421, 1915-3p, and 4488 define STAMPEDE subjects in group 1 compared with controls. Group 1: STAMPEDE subjects 16, 18, 13, 17, 15, 21, 12, 23, 14, & 11; Control: all 15 control subjects; Control + others: controls plus STAMPEDE subjects exclusive of group 1. [file 1755-8794-7-58-S11.pdf]

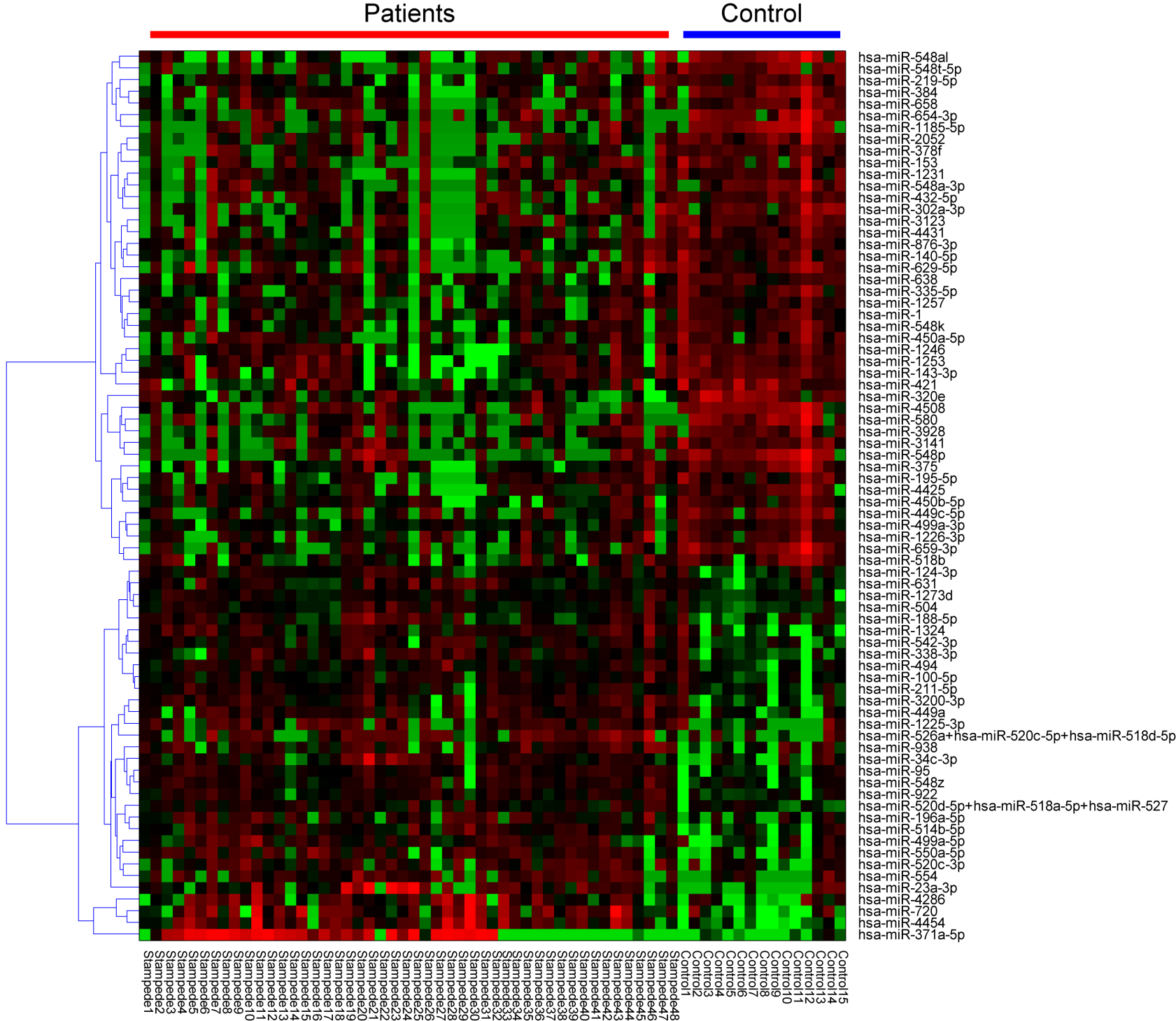


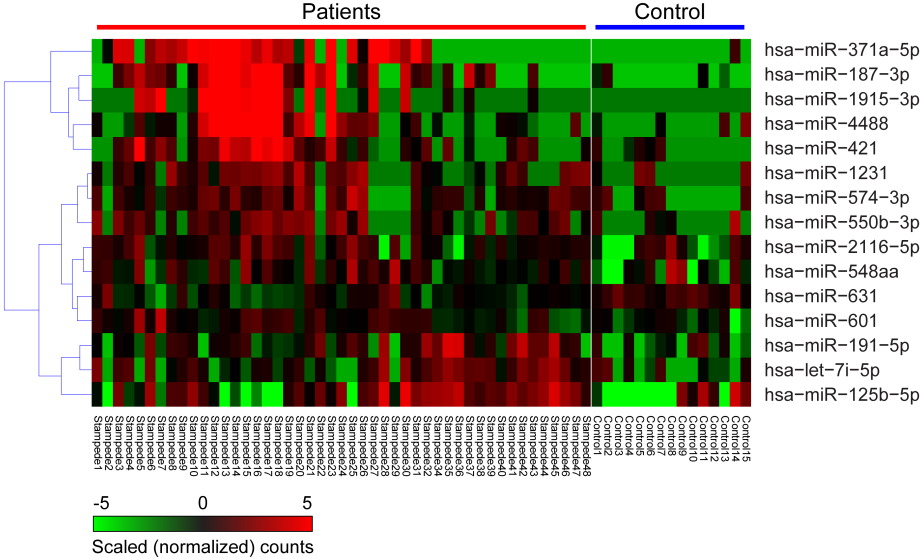

Supplement: Additional file 12 — One way cluster analysis of 76 differentially expressed miRNAs in urine. [file 1755-8794-7-58-S12.docx]

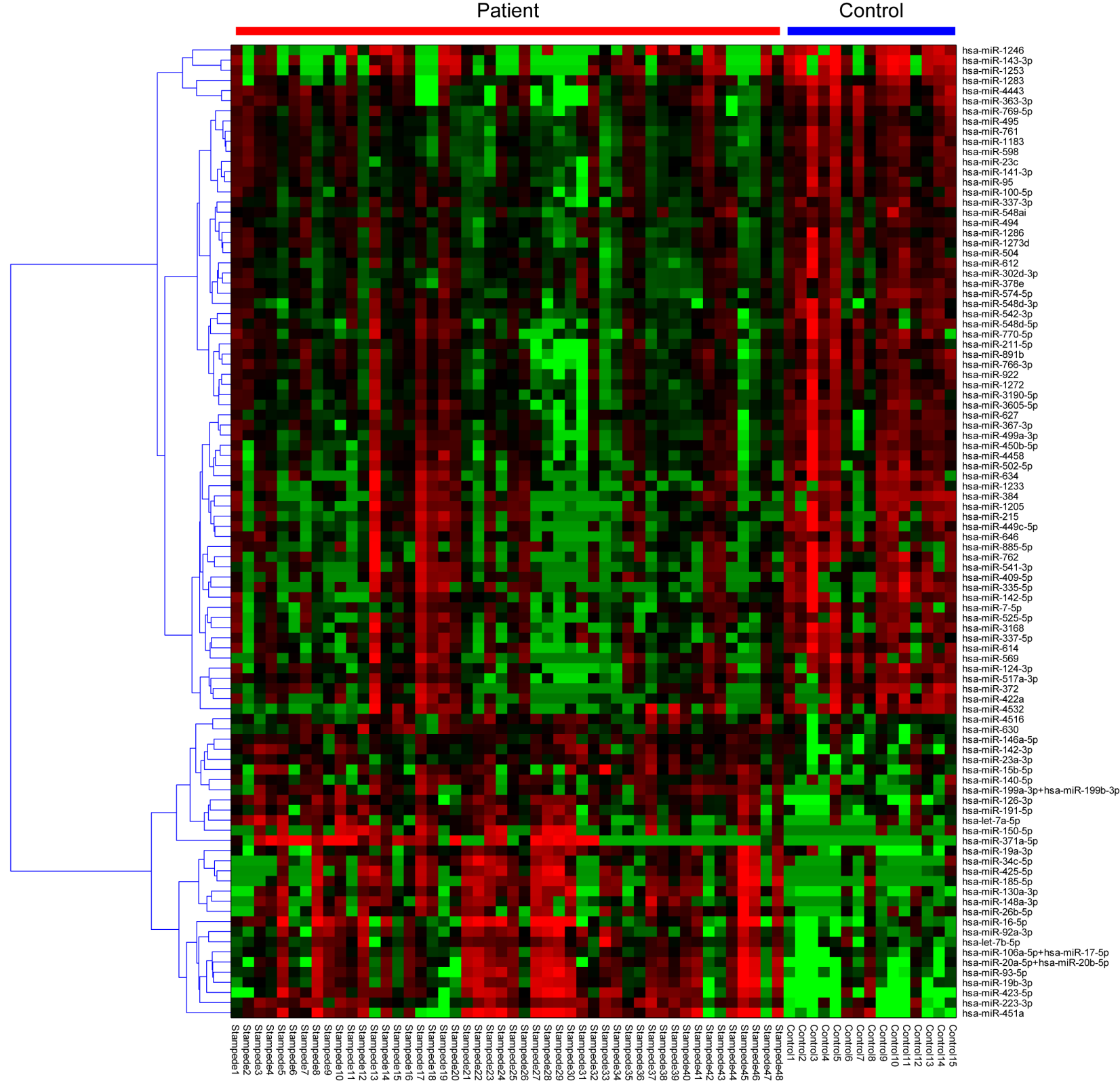

Supplement: Additional file 13 — One way cluster analysis of 96 differentially expressed miRNAs in serum. [file 1755-8794-7-58-S13.docx]

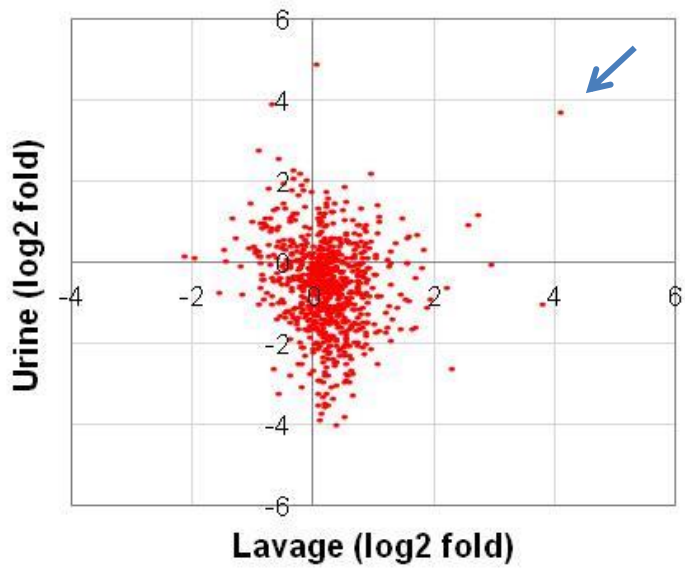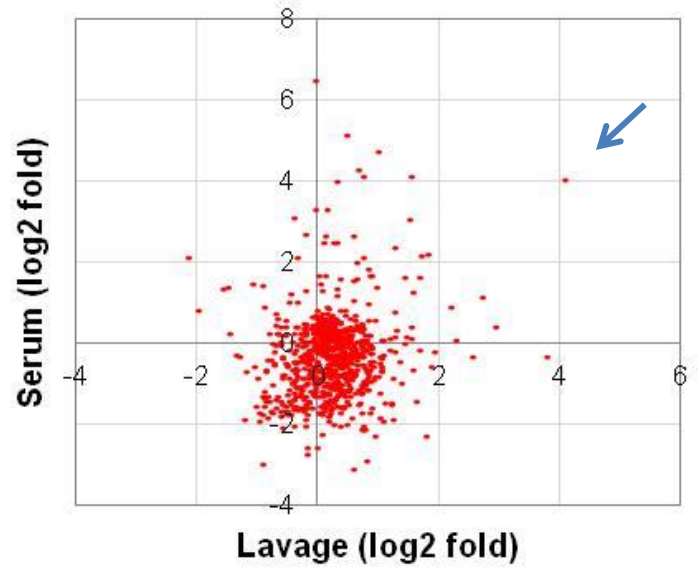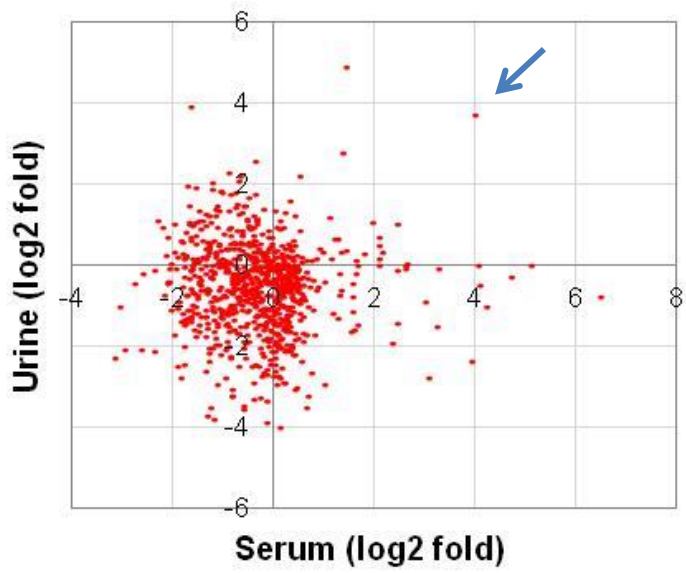

Supplement: Additional file 14 — Pair-wise expression of miRNA in BAL, urine, or serum. Upper left, the average expression of a specific miRNA (as the log2 level) in urine plotted as a function of its average levels in BAL; Upper right, miRNA expression in serum as a function of the levels in BAL; Lower left, miRNA expression in urine as a function of the levels in serum. The blue arrow identifies the expression of miRNA 371a-5p which was elevated in all three samples from STAMPEDE subjects. While the majority of the miRNA epression changes in BAL, urine, and serum were independent of one another, miRNA 371a-5p was consistently overexpressed in BAL, urine, and serum from STAMPEDE subjects. [file 1755-8794-7-58-S14.pdf]

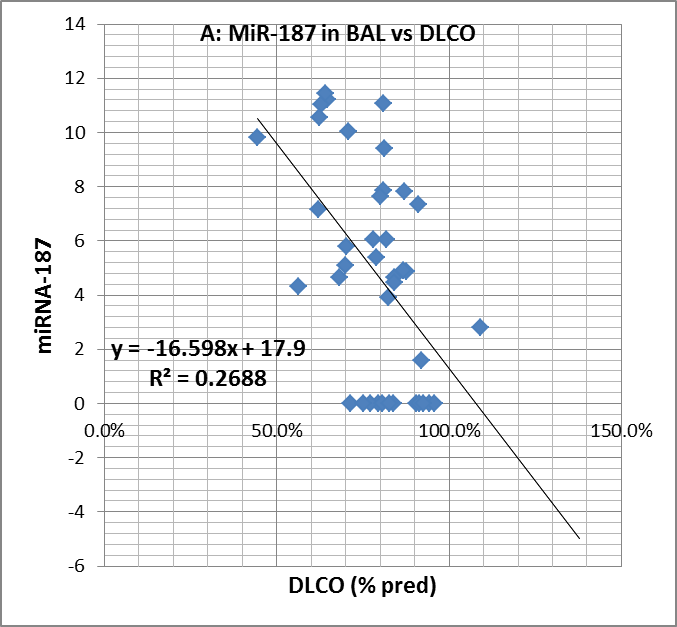

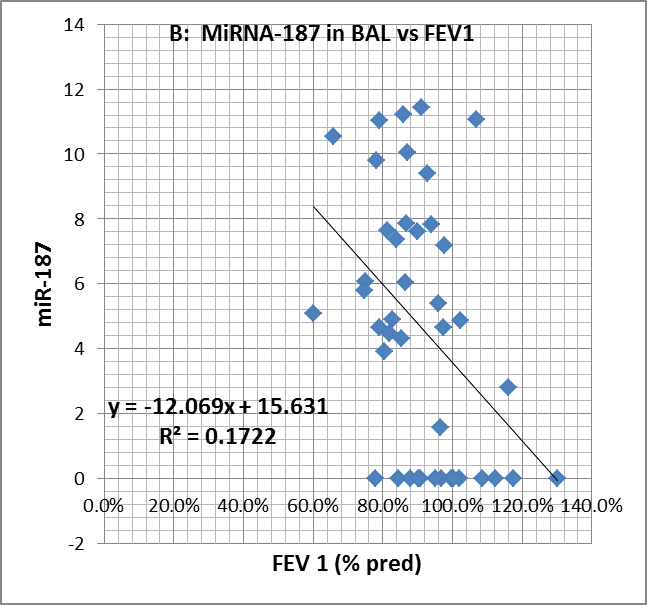

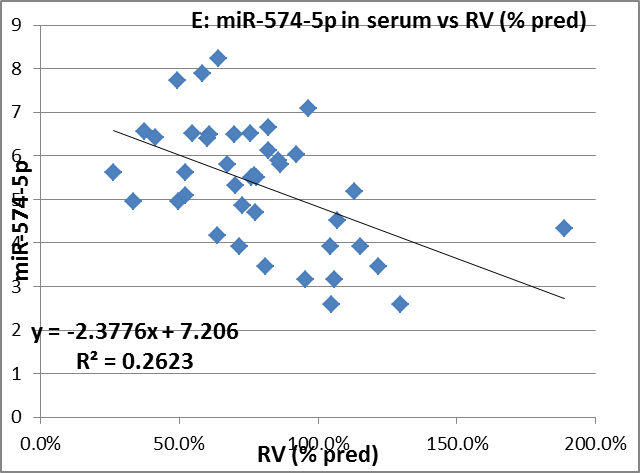

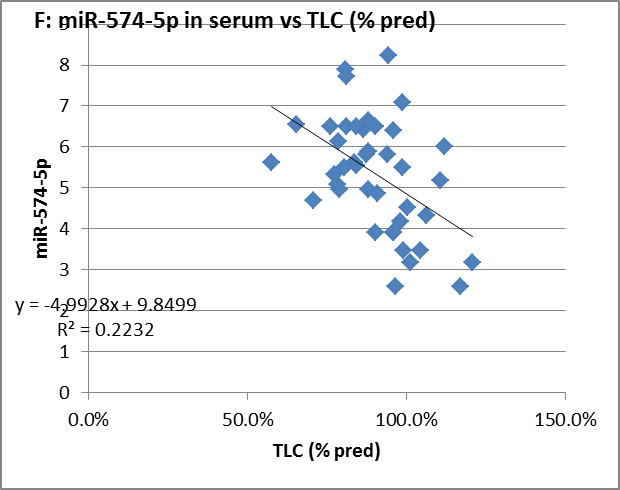

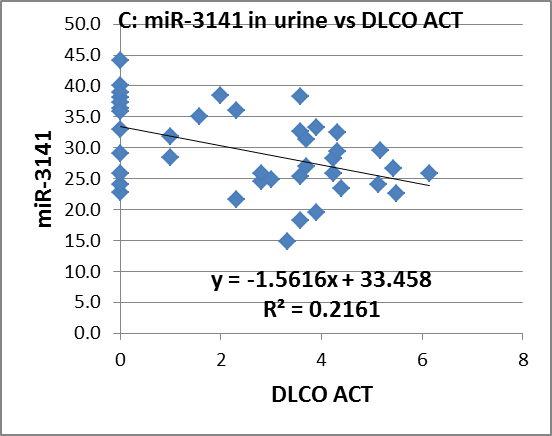

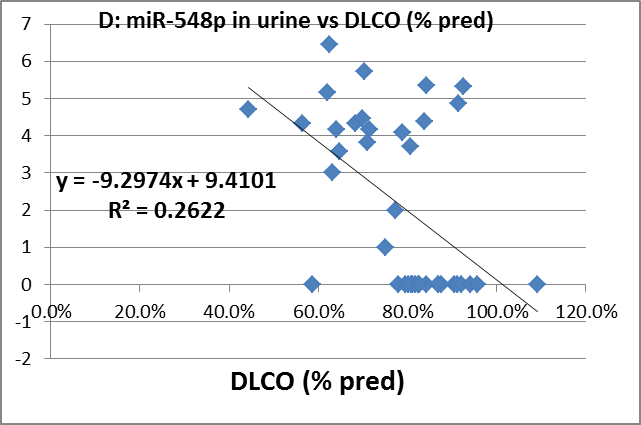

Supplement: Additional file 15 — Plots of selected miRNAs that showed associations with medical chart parameters by calculation of Pearson correlations. [file 1755-8794-7-58-S15.docx]
