# Supplementary material for: Protein and microRNA biomarkers from lavage, urine, and serum in military personnel evaluated for dyspnea
Source: BMC Med Genomics. 2014 Oct 5;7:58. doi: 10.1186/1755-8794-7-58 (PMC4193960; doi:10.1186/1755-8794-7-58)

Additional file 15

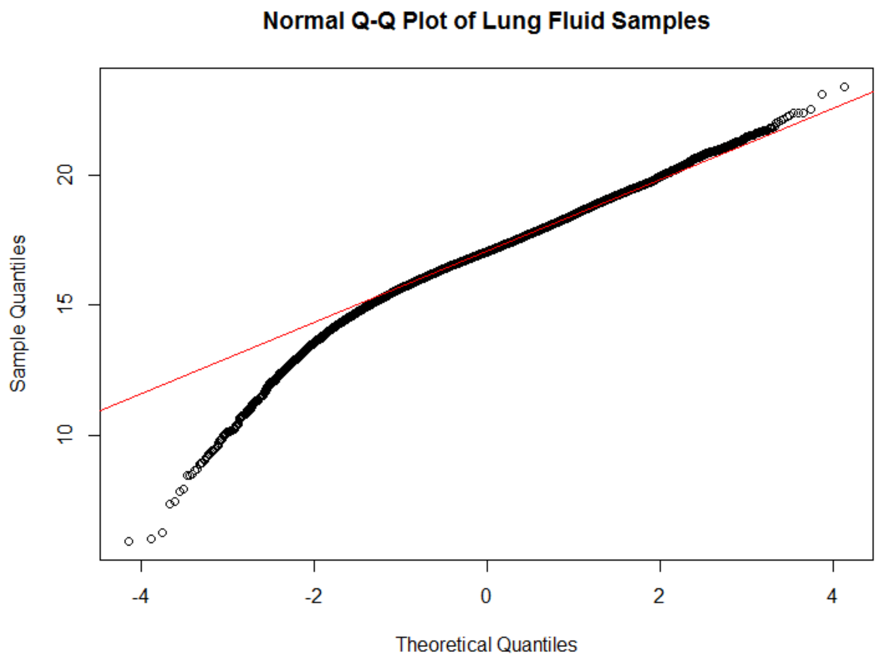

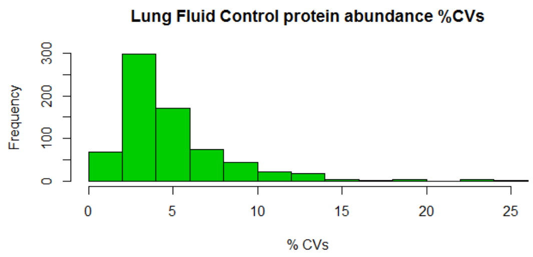

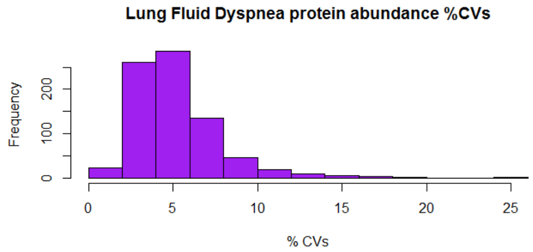

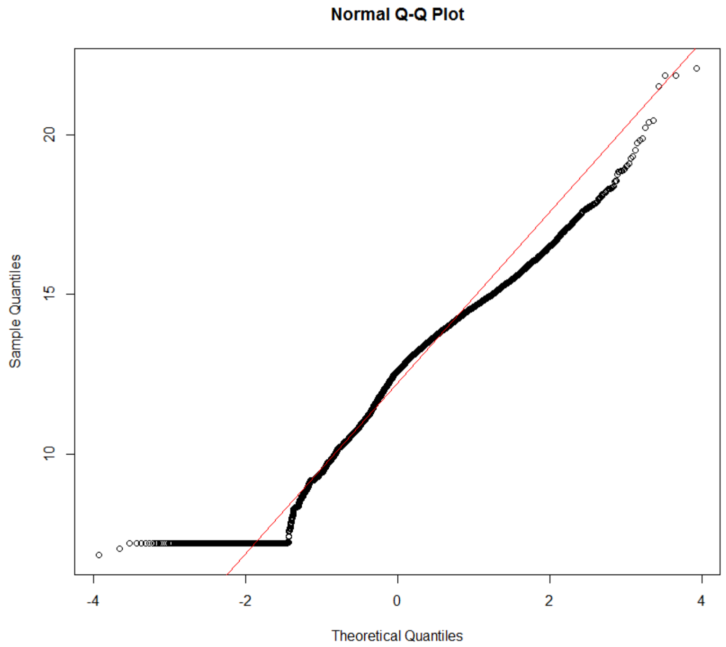

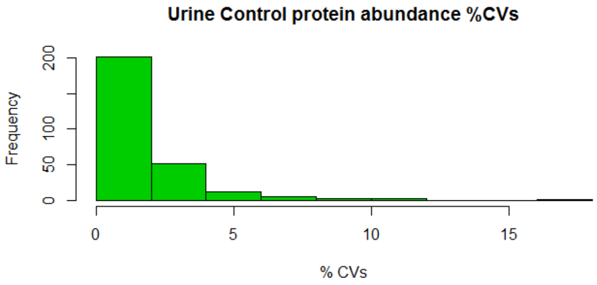

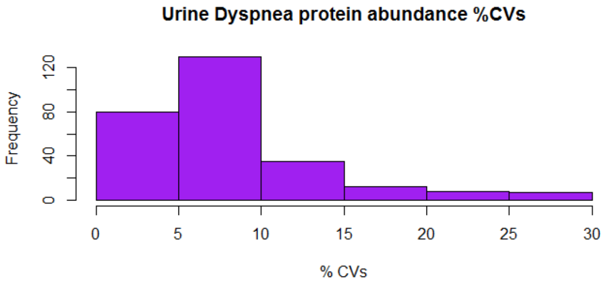

Supplement: Additional file 4 — Abundance and variance distributions for BAL and urine proteins. (Upper left): normal quantile (Q-Q) plot of proteins identified from BAL fluid; (Upper right): variance distributions for BAL proteins from control subjects (green) or STAMPEDE subjects (violet); (Lower left): normal Q-Q plot of proteins identified from urine; (lower right): variance distributions for urine proteins from control subjects (green) or STAMPEDE subjects (violet). [file 1755-8794-7-58-S4.pdf]
