# Supplementary material for: Protein and microRNA biomarkers from lavage, urine, and serum in military personnel evaluated for dyspnea
Source: BMC Med Genomics. 2014 Oct 5;7:58. doi: 10.1186/1755-8794-7-58 (PMC4193960; doi:10.1186/1755-8794-7-58)
Supplement: Additional file 8 — Proteins reliably identified in common from BAL (n = 79) and urine (n = 74) from Additional files3&4 are listed by their UniProt accession identifiers. The proteins in common from BAL and urine are listed as well. [file 1755-8794-7-58-S8.docx]

|  | BAL proteins | Urine proteins | Common |
| --- | --- | --- | --- |
| 1 | FABP4 | F198B | TRY6 |
| 2 | LAC2 | KV309 | KV404 |
| 3 | KV206 | TSP1 | K2C1 |
| 4 | IGHG4 | FLNA | HPT |
| 5 | TRY6 | DEF3 | FETUA |
| 6 | HIS3 | SAP3 | TFF3 |
| 7 | ACE | IGSF8 |  |
| 8 | A1AT | LAIR2 |  |
| 9 | KV404 | HEPC |  |
| 10 | LV605 | PVR |  |
| 11 | ICAM1 | KV404 |  |
| 12 | A1AG2 | CATB |  |
| 13 | LAC3 | APOD |  |
| 14 | K2C1 | COL12 |  |
| 15 | HPT | CYTM |  |
| 16 | LUM | DEFB1 |  |
| 17 | ANT3 | REG1A |  |
| 18 | AACT | WISP2 |  |
| 19 | A1AG1 | ACV1B |  |
| 20 | A1BG | FBLN3 |  |
| 21 | HEMO | KV205 |  |
| 22 | APOA2 | ENDD1 |  |
| 23 | APOA1 | LAIR1 |  |
| 24 | PXP | YIPF3 |  |
| 25 | A2MG | ZA2G |  |
| 26 | HBD | CD59 |  |
| 27 | HBA | FETUA |  |
| 28 | HBB | PTGDS |  |
| 29 | S10A6 | HPT |  |
| 30 | CAH2 | CAD13 |  |
| 31 | TCPZ | CD14 |  |
| 32 | DPY30 | AMBP |  |
| 33 | APT | VGF |  |
| 34 | S38AA | THY1 |  |
| 35 | KNG1 | GNS |  |
| 36 | ANGI | CSF1 |  |
| 37 | AFAM | PLMN |  |
| 38 | CFAB | IBP7 |  |
| 39 | FETUA | TFF3 |  |
| 40 | HSP71 | SLUR2 |  |
| 41 | HSP77 | SLAF5 |  |
| 42 | RADI | DAF |  |
| 43 | TFF3 | PGBM |  |
| 44 | MUC5A | XYLT1 |  |
| 45 | TBA3E | HMCN1 |  |
| 46 | GSTA5 | BT2A3 |  |
| 47 | SPB4 | CD44 |  |
| 48 | SPB3 | SHSA5 |  |
| 49 | BASP1 | MATN4 |  |
| 50 | IDHC | WFDC2 |  |
| 51 | HS90A | CYTC |  |
| 52 | GELS | CD248 |  |
| 53 | S10A4 | ROBO4 |  |
| 54 | TYB4 | CADM4 |  |
| 55 | PGK1 | LMAN2 |  |
| 56 | UBA1 | CUBN |  |
| 57 | GSHR | QPCT |  |
| 58 | TRXR1 | TRY6 |  |
| 59 | MT2 | PPAP |  |
| 60 | PAIRB | KLK1 |  |
| 61 | VIME | CD99 |  |
| 62 | AL3A1 | CADH2 |  |
| 63 | ADH7 | TRY3 |  |
| 64 | AK1C4 | UROM |  |
| 65 | MVP | K2C1 |  |
| 66 | IGHD | HORN |  |
| 67 | CYTT | MT3 |  |
| 68 | CYTS | CO3A1 |  |
| 69 | AMY2B | LYSC |  |
| 70 | LCN1 | SCG1 |  |
| 71 | BPIB1 | PP1A |  |
| 72 | CF058 | SBSN |  |
| 73 | SLPI | SFTA1 |  |
| 74 | MUC5B | UTER |  |
| 75 | DMBT1 |  |  |
| 76 | TRFL |  |  |
| 77 | SFTPD |  |  |
| 78 | PSPB |  |  |
| 79 | IC1 |  |  |
