# Supplementary material for: Protein and microRNA biomarkers from lavage, urine, and serum in military personnel evaluated for dyspnea
Source: BMC Med Genomics. 2014 Oct 5;7:58. doi: 10.1186/1755-8794-7-58 (PMC4193960; doi:10.1186/1755-8794-7-58)
Supplement: Supplementary file 5 [file 1755-8794-7-58-S5.docx]

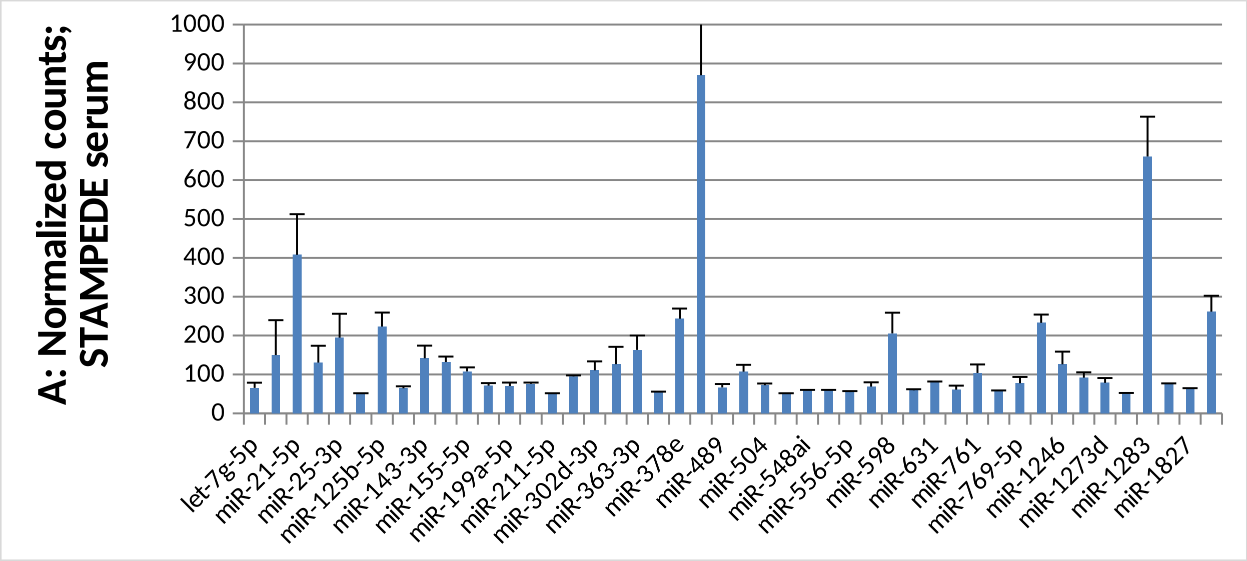
A: Normalized counts; STAMPEDE serum
0
100
200
300
400
500
600
700
800
900
1000
let-7g-5p
miR-21-5p
miR-25-3p
miR-125b-5p
miR-143-3p
miR-155-5p
miR-199a-5p
miR-211-5p
miR-302d-3p
miR-363-3p
miR-378e
miR-489
miR-504
miR-548ai
miR-556-5p
miR-598
miR-631
miR-761
miR-769-5p
miR-1246
miR-1273d
miR-1283
miR-1827

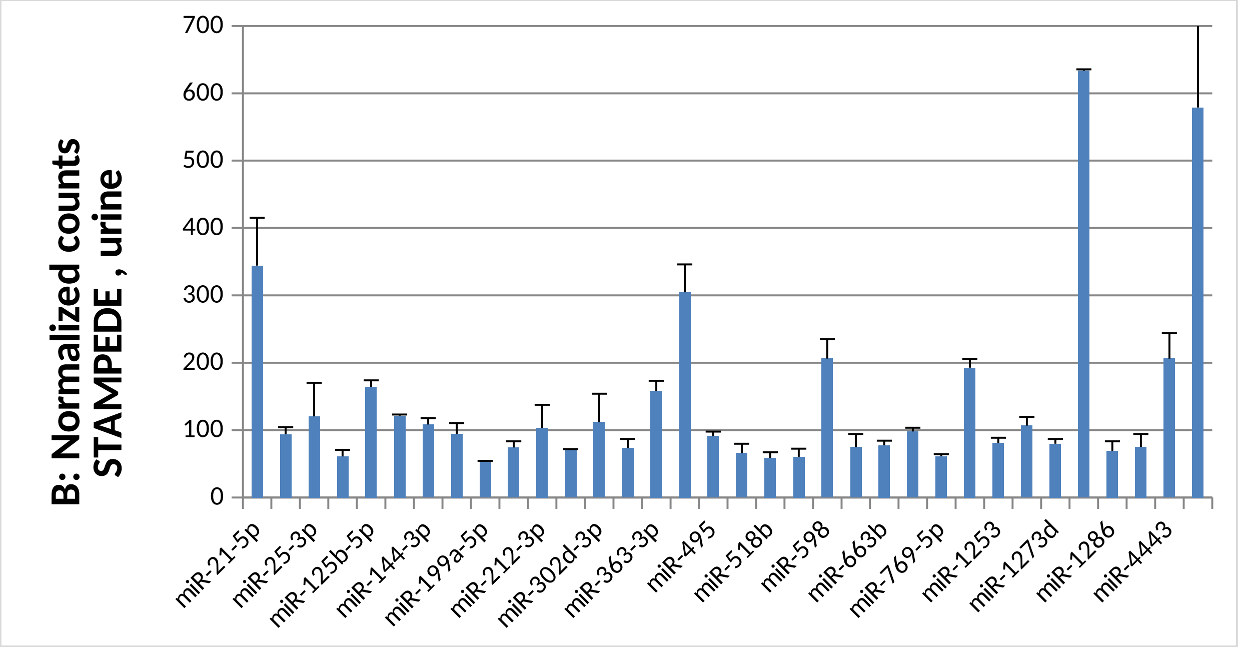
B: Normalized counts STAMPEDE , urine
0
100
200
300
400
500
600
700
miR-21-5p
miR-25-3p
miR-125b-5p
miR-144-3p
miR-199a-5p
miR-212-3p
miR-302d-3p
miR-363-3p
miR-495
miR-518b
miR-598
miR-663b
miR-769-5p
miR-1253
miR-1273d
miR-1286
miR-4443

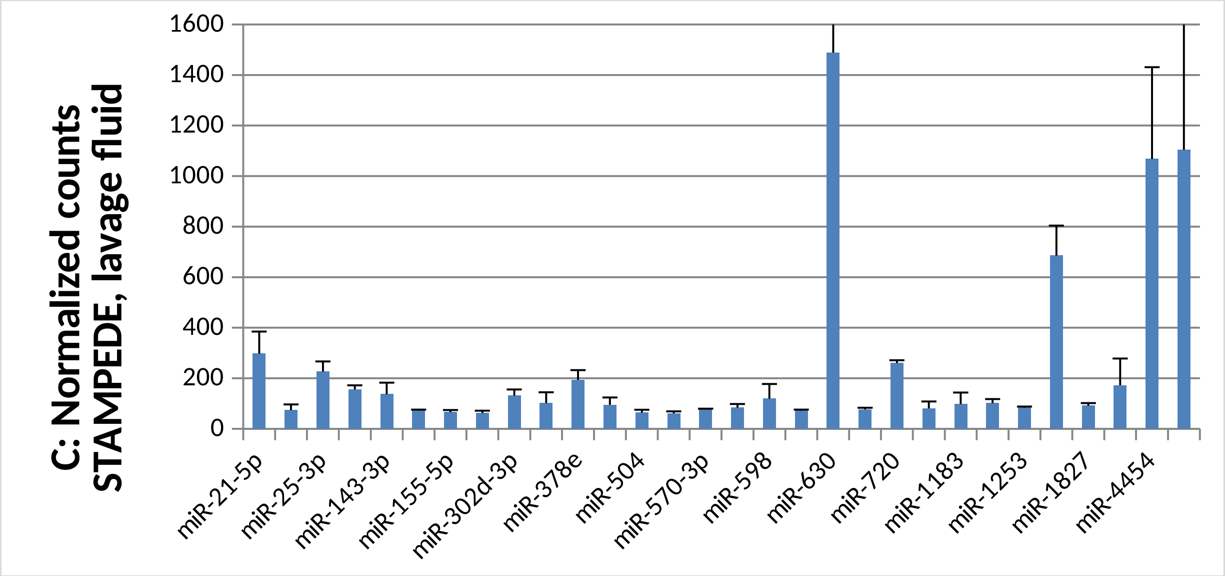
C: Normalized counts STAMPEDE, lavage fluid
0
200
400
600
800
1000
1200
1400
1600
miR-21-5p
miR-25-3p
miR-143-3p
miR-155-5p
miR-302d-3p
miR-378e
miR-504
miR-570-3p
miR-598
miR-630
miR-720
miR-1183
miR-1253
miR-1827
miR-4454
