# Supplementary material for: Protein and microRNA biomarkers from lavage, urine, and serum in military personnel evaluated for dyspnea
Source: BMC Med Genomics. 2014 Oct 5;7:58. doi: 10.1186/1755-8794-7-58 (PMC4193960; doi:10.1186/1755-8794-7-58)
Supplement: Supplementary file 6 [file 1755-8794-7-58-S6.docx]

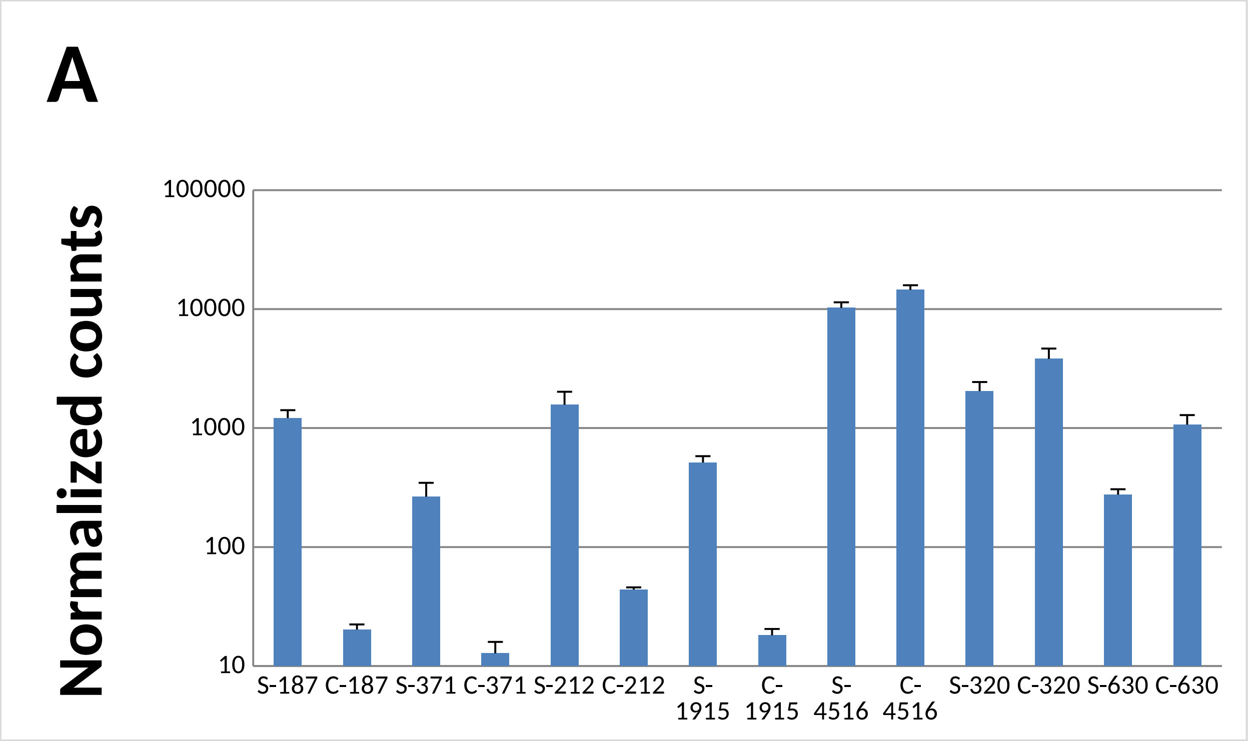
A
Normalized counts
100000
10000
1000
100
10
S-187
C-187
S-371
C-371
S-212
C-212
S-1915
C-1915
S-4516
C-4516
S-320
C-320
S-630
C-630

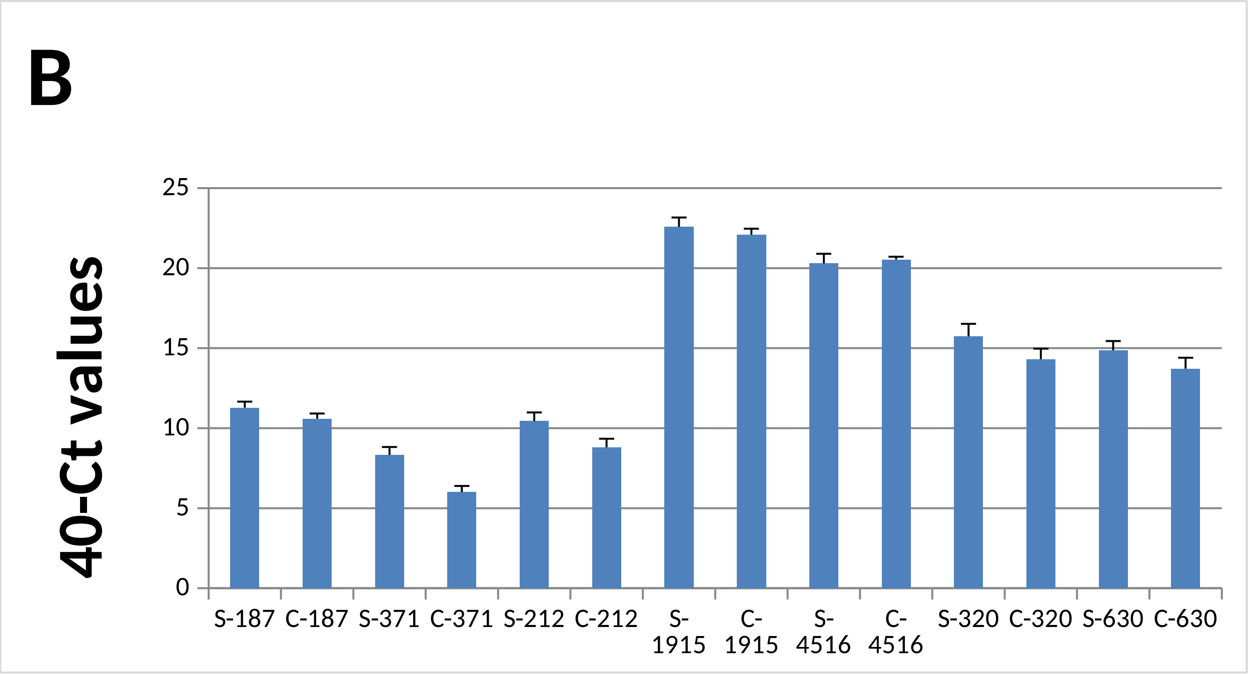
B
40-Ct values
25
20
15
10
5
0
S-187
C-187
S-371
C-371
S-212
C-212
S-1915
C-1915
S-4516
C-4516
S-320
C-320
S-630
C-630
